# Supplementary material for: Acceptability and tolerability of repeated intramuscular electroporation of Multi-antigenic HIV (HIVMAG) DNA vaccine among healthy African participants in a phase 1 randomized controlled trial
Source: PLoS One. 2020 May 29;15(5):e0233151. doi: 10.1371/journal.pone.0233151 (PMC7259687; doi:10.1371/journal.pone.0233151)
Supplement: S1 File — (PDF) [file pone.0233151.s002.pdf]

Protocol Title:	A Phase 1 Double-Blind, Randomized, Placebo-Controlled Trial to Evaluate the Safety and Immunogenicity of a Multiantigen HIV (HIV-MAG) plasmid DNA (pDNA) Vaccine co-administered with Recombinant Human IL-12 pDNA (GENEVAX® IL-12) followed or preceded by Recombinant Ad35-GRIN/ENV HIV Vaccine in HIV-Uninfected, Healthy Volunteers
Protocol Number:	**IAVI B004**
Phase:	**Phase 1**
IND Number:	
Sponsor:	International AIDS Vaccine Initiative (IAVI)<br>125 Broad Street, 9th Floor<br>New York, New York 10002<br>USA
Sponsor Status:	**Non-Profit Organization**
Collaborating Companies:	Profectus Biosciences, Inc.<br><br>Ichor Medical Systems, Inc.
Date of Protocol Version 1.0:	30 June 2011

SYNOPSIS

**TITLE:**	A Phase 1 Double-Blind, Randomized, Placebo-Controlled Trial to Evaluate the Safety and Immunogenicity of a Multiantigen HIV (HIV-MAG) plasmid DNA (pDNA) Vaccine co-administered with Recombinant Human IL-12 pDNA (GENEVAX® IL-12) followed or preceded by Recombinant Ad35-GRIN/ENV HIV Vaccine in HIV-Uninfected, Healthy Volunteers
**PROTOCOL NUMBER:**	B004
**PHASE:**	Phase 1
**SPONSOR:**	International AIDS Vaccine Initiative (IAVI)<br>125 Broad Street, 9th Floor<br>New York, New York 10002, USA
**Sponsor Status:**	Not for Profit Organization
**STUDY VACCINES / INVESTIGA-TIONAL PRODUCTS**	• HIV-MAG: A plasmid DNA vaccine consisting of two DNA plasmids mixed at the time of administration:<br>o ProfectusVax HIV-1 subtype B *gag/pol* DNA plasmid<br>o ProfectusVax HIV-1 subtype B *nef/tat/vif, env* (subtype B primary isolate Env gp160) DNA plasmid<br><br>• GENEVAX® IL-12: A DNA plasmid encoding the p35 and p40 subunits of human IL-12.<br><br>• Ad35-GRIN/ENV: A recombinant replication-incompetent Adenovirus serotype 35 vector containing HIV-1 subtype A *gag, reverse transcriptase, integrase* and *nef* gene sequences (GRIN) and a recombinant replication-incompetent Adenovirus serotype 35 expressing HIV-1 subtype A gp140 *env* gene (ENV)<br><br>• Placebo: Sodium Chloride Injection, USP 0.9%
**OBJECTIVES:**	***Primary:***<br><br>1. To evaluate the safety and tolerability of HIV-MAG with or without co-administered GENEVAX® IL-12 given intramuscularly by *in vivo* electroporation (IM/EP) using the Ichor Medical Systems TriGrid™ Delivery System (TDS-IM) followed by Ad35-GRIN/ENV in each of four different regimens<br><br>2. To evaluate the safety and tolerability of Ad35-GRIN/ENV followed by HIV-MAG with co-administered GENEVAX® IL-12 given intramuscularly by *in vivo* electroporation (IM/EP) using the Ichor Medical Systems TriGrid™ Delivery System (TDS-IM)

	Secondary<br><br>1. To determine whether the HIV-specific immune responses induced by HIV-MAG are increased by co-administration of GENEVAX® IL-12 in a prime-boost regimen with Ad35-GRIN/ENV given as boost and if so, which dosage level induces higher immune responses<br><br>2. To compare HIV-specific immune responses induced by HIV-MAG co-administered with GENEVAX® IL-12 IM/EP once at M0 followed by Ad35-GRIN/ENV IM at M4 with those induced by HIV-MAG co-administered with GENEVAX® IL-12 IM/EP at M0,1,2 followed by Ad35-GRIN/ENV IM at M6<br><br>3. To compare HIV-specific immune responses induced by Ad35-GRIN/ENV IM prime at M0 followed by HIV-MAG co-administered with GENEVAX® IL-12 IM/EP boost at M4 with those induced by HIV-MAG co-administered with GENEVAX® IL-12 IM/EP prime at M0 followed by Ad35-GRIN/ENV IM boost at M4
**ENDPOINTS**	***Primary:***<br><br>*Safety and Tolerability:*<br>To assess safety and tolerability of the different prime-boost regimens:<br><br>1. Proportion of volunteers with moderate or greater reactogenicity (i.e., solicited adverse events) during a 7-day follow-up period after each vaccination<br><br>2. Proportion of volunteers with moderate or greater and/or vaccine-related unsolicited adverse events (AEs), including safety laboratory (biochemical, haematological) parameters, from the day of each vaccination up to 28 days post each vaccination<br><br>3. Proportion of volunteers with vaccine-related serious adverse events (SAEs) throughout the study period<br><br>***Secondary:***<br><br>*Immunogenicity:*<br>To assess (qualitative and quantitative) immune responses elicited by the different prime-boost regimens:<br><br>1. Proportion of volunteers with HIV-1 specific T-cell responses quantified by IFN-γ ELISPOT and magnitude of responses<br><br>2. Frequency and magnitude of polyfunctional HIV-1 specific CD4+ or CD8+ T-cell responses assessed by Intracellular Cytokine Staining and Polychromatic Flow Cytometry (ICS/PFC) measuring

	a panel of cytokines, functional and phenotypic markers after stimulation with HIV-1 antigen peptide pools<br><br>3. Epitope mapping to determine specificity and breadth of the responses<br><br>***Exploratory:***<br><br>*Immunogenicity:*<br>Additional immunogenicity assessments may include:<br>1. Binding antibodies to HIV antigens (frequency and magnitude)<br>2. Neutralizing antibodies to HIV antigens (seropositivity rates and magnitude of antibody titres)<br>3. Neutralizing antibodies to the Ad35 vector (frequency and magnitude)<br><br>Responses may be further characterized to include:<br>1. Ability of CD8 cells to inhibit HIV replication in a Viral Inhibition Assay (VIA)<br>2. Capacity of antigen specific T cells to proliferate<br>3. Flow cytometry to assess markers for memory, exhaustion and activation, and secretion of cytokines other than IFN-γ, IL-2 and TNF-α<br>4. Repertoire analysis to assess B cell response<br><br>*Serum antibodies against human IL-12:*<br>1. Proportion of volunteers/vaccine recipients who develop antibodies against human IL-12<br><br>*EP Tolerability:*<br>1. Proportion of volunteers judging the EP procedure as acceptable

STUDY DESIGN

		Months 0, 1, 2	Month 6
**Study Groups**	**N vaccine / placebo**	**Prime Vaccine (dosage, delivery)**	**Boost Vaccine (dosage, delivery)**
1	**12/3**	HIV-MAG (3,000mcg) (IM/EP*)	Ad35-GRIN/ENV (2x10^{10}vp***, IM**)
2	**12/3**	HIV-MAG (3,000mcg) + GENEVAX® IL-12 (100mcg) (IM/EP)	Ad35-GRIN/ENV (2x10^{10} vp, IM)
3	**12/3**	HIV-MAG (3,000mcg) + GENEVAX® IL-12 (1000mcg) (IM/EP)	Ad35-GRIN/ENV (2x10^{10} vp, IM)
		**Month 0**	**Month 4**
4	**12/3**	HIV-MAG (3,000mcg) + GENEVAX® IL-12 (1000mcg) (IM/EP)	Ad35-GRIN/ENV (2x10^{10} vp, IM)
5	**12/3**	Ad35-GRIN/ENV (2x10^{10} vp, IM)	HIV-MAG (3,000mcg) + GENEVAX® IL-12 (1000mcg) (IM/EP)
**Total**	**75 (60/15)**		

*IM/EP=intramuscular by *in vivo* electroporation
**IM=intramuscularly
*** vp=viral particles

METHODS

See Schedule of Procedures; Appendix A (Groups 1-3), Appendix B (Groups 4-5)

STUDY POPULATION

Healthy male or female adults, 18 to 50 years of age, who do not report high-risk behaviour for HIV infection, who are available for the duration of the trial, who are willing to undergo HIV testing, use an effective method of contraception, and who, in the opinion of the principal investigator or designee, understand the study and who provide written informed consent.

Principal exclusion criteria include confirmed HIV infection, pregnancy and lactation, significant acute or chronic disease, clinically significant laboratory abnormalities, recent vaccination or receipt of a blood product, previous receipt of an HIV vaccine, and previous severe local or systemic reactions to vaccination or history of severe allergic reactions.

NUMBER OF VOLUNTEERS:

Approximately 75 volunteers (60 vaccine/15 placebo recipients) will be included in the study. An over-enrolment of up to 10% (up to 83 volunteers total) will be permitted in the study to facilitate rapid enrolment.

FORMULATIONS, VOLUMES and ROUTES of INJECTIONS,

HIV-MAG vaccine consisting of two DNA plasmids (ProfectusVax HIV-1 *gag/pol* DNA plasmid and ProfectusVax HIV-1 *nef/tat/vif, env* DNA plasmid) are formulated in 30mM citrate buffer (pH 6.5) containing 150 mM NaCl, 0.01% EDTA, and 0.25% bupivacaine HCl in separate vials. The two plasmids are mixed immediately prior to administration.

GENEVAX® IL-12 is formulated in 30 mM citrate buffer (pH 6.5) containing 150 mM NaCl, 0.01% EDTA and 0.25% bupivacaine HCl. GENEVAX® IL-12 is mixed with the HIV-MAG immediately prior to administration.

All administrations of HIV-MAG with GENEVAX® IL-12 (or placebo) consist of 2 IM injections, one into each medial deltoid, by *in vivo* electroporation (EP) using the Ichor Medical Systems TriGrid™ Delivery System (TDS-IM).

Ad35-GRIN/ENV containing two Ad35 constructs (Ad35-GRIN and Ad35-ENV) are co-formulated in Tris 10 mM pH 8.5, Sucrose 342.3 g/L, 1mM $MgCl_2$, Tween80 54 mg/L and 150mM NaCl in the same vial. All administrations of Ad35-GRIN/ENV (or placebo) consist of one IM injection in the deltoid muscle.

Vaccine/<br>Placebo	Dosage Level(s)	Total Injected Volume (per site)	Route of Administration
HIV-MAG (alone)	3,000 mcg	1.0 mL (2x0.5 mL)	IM/EP
HIV-MAG+<br>GENEVAX® IL-12	HIV-MAG (3000 mcg)<br>+<br>GENEVAX® IL-12<br>(100 mcg)	1.2 mL (2x0.6 mL)	IM/EP
	HIV-MAG (3000 mcg)<br>+<br>GENEVAX® IL-12<br>(1000mcg)	1.5 mL (2x0.75 mL)	
Ad35-GRIN/ENV	$2x10^{10}$ vp	0.5 mL	IM
Placebo	N/A	1.0 mL (2x0.5 mL)<br>1.2 mL (2x0.6 mL)<br>1.5 mL (2x0.75 mL)	IM/EP
Placebo	N/A	0.5 mL	IM

DURATION OF STUDY PARTICIPA-TION:

Volunteers will be screened up to 42 days before the 1st vaccination and will be followed for 12 months after the first vaccine administration. It is anticipated that it will take approximately 3 months to enrol the study.

The anticipated study duration for each volunteer is approximately 13 months from screening through last study visit.

RANDOMIZA-TION and BLINDING

Volunteers will be randomly assigned to one of five groups described in the study design table above.

Study site staff and volunteers will be blinded in terms of vaccine versus placebo and GENEVAX® IL-12 dosage levels, but not to schedule, number of vaccinations administered and delivery method. An unblinded study pharmacist at each site will be responsible for vaccine preparation and accountability.

**EVALUATION FOR INTER-CURRENT HIV INFECTION:**	Volunteers will be tested for HIV according to the Schedule of Procedures. Test results will be interpreted according to a pre-determined diagnostic algorithm. Should one or more serological HIV test(s) post-vaccination be positive, a nucleic-acid-based HIV test will be performed to distinguish a true HIV infection acquired through exposure in the community from HIV seropositivity due to the vaccine-induced antibody response. HIV testing at additional time points may be performed upon request of the volunteer and principal investigator or designee as medical or social circumstances warrant.<br><br>To maintain blinding of study staff with direct contact with the volunteers, HIV test results will be reported to the clinical team as "HIV-uninfected" or as "HIV-infected" (*ONLY* once intercurrent HIV infection is confirmed).
**SAFETY MONITORING AND STATISTICAL CONSIDERA-TIONS:**	All clinical trial data collected, identified only by a volunteer identification number, will be entered into a database.<br><br>Safety will be continually monitored by the Investigators, the Sponsor's Medical Monitor and a Protocol Safety Review Team (PSRT); detailed stopping criteria are pre-defined.<br><br>All safety data will be reviewed by an independent Safety Review Board (SRB) once the first 15 volunteers randomized across all groups have reached the M 2.5 and M 6.5 time points.<br><br>*Ad-hoc* safety review may be specifically requested by the Sponsor, Profectus, Ichor, the Principal Investigators, Ethics Committees, Regulatory Authorities, or by the SRB.<br><br>At the end of the study, a full analysis will be prepared according to a pre-specified statistical analysis plan. In addition, there may also be interim reviews of blinded data. All clinical and routine laboratory data will be included in the safety analysis. Immunogenicity analysis will be performed according to a predefined analysis plan for all volunteers who received vaccine or placebo.

TABLE OF CONTENTS

2.0 SIGNATURE PAGE 13

3.0 INTRODUCTION AND BACKGROUND INFORMATION 14

3.1 Study Rationale 16
3.2 Experience with HIV-MAG 17
3.3 Experience with recombinant human IL-12 pDNA as molecular adjuvant for HIV pDNA vaccines 17
3.4 Experience with Ichor TriGrid™Delivery System for in vivo electroporation 18
3.5 Experience with Ad35-GRIN/ENV 19

4.0 STUDY OBJECTIVES 20

4.1 Primary Objectives 20
4.2 Secondary Objectives 20

5.0 STUDY ENDPOINTS AND STUDY DESIGN 20

5.1 Study Endpoints 20
5.1.1 Primary Endpoints 20
5.1.2 Secondary Endpoints 21
5.1.3 Exploratory Endpoints 21
5.2 Study Design 21
Table 5.2-1: Study Design 22
5.2.1 Duration of the Study 22
5.2.2 Study Population 22
5.2.3 Inclusion Criteria 23
5.2.4 Exclusion Criteria 23
5.2.5 Recruitment of Volunteers 25

6.0 STUDY VISITS 25

6.1 Screening Period 25
6.2 Vaccination Visits 26
6.3 Post-Vaccination Visits 27
6.4 Additional Follow-up Visits 27
6.5 Unscheduled Visits 27
6.6 Final Study Visit/Early Termination Visit 28

7.0 STUDY PROCEDURES 28

7.1 Informed Consent Process 28
7.2 Medical History and Physical Examination 29
7.3 HIV Testing and HIV-test Counselling 29
7.4 HIV Risk Reduction Counselling 29
7.5 Family Planning Counselling 30
7.6 Specimens 30
7.7 Reimbursement 30
7.8 Randomization and Blinding 30
7.9 Unblinding Procedure for Individual Volunteers 31
7.10 Referral to Long Term Follow-Up Study 31

8.0 INVESTIGATIONAL PRODUCT 31

8.1 Description 31
8.1.1 HIV-MAG 31
8.1.2 Ad35-GRIN/ENV 32
8.1.3. GENEVAX® IL-12 32
8.1.4. Placebo 32

Table 8.1.4-1: Formulation of Investigational Products 33
8.1.5. Ichor Medical Systems TriGrid™ Delivery System (TDS-IM) 33
8.2 Shipment and Storage 34
8.3 Preparation of Investigational Product (IP) 35
8.4 Administration of Investigational Product 35
8.5 Accountability and Disposal of Investigational Product 35

9.0 ASSESSMENTS 35

9.1 Safety Assessments 35
9.1.1 Local reactogenicity 36
9.1.2 Systemic reactogenicity 36
9.1.3 Vital signs 36
9.1.4 Other adverse events 36
9.1.5 Concomitant Medications 36
9.1.6 Routine laboratory parameters 37
Table 9.1.6-1: Laboratory Parameters 37
9.1.7 Specific screening tests: 37
9.2 Immunogenicity Assessments 38
9.2.1 Antibody Responses 38
9.2.2 Cellular Responses 38
9.2.3 PBMC, Serum and Plasma Storage 38
9.3 Other Assessments 38
9.3.1 HLA Typing 38
9.3.2 Serum Antibodies against Human IL-12 39
9.3.3 HIV test 39
9.3.4 Pregnancy Test 39
9.3.5 Screening Assessment 39
9.3.6 HIV Risk Assessment 39
9.3.7 Electroporation Tolerability Assessment 39
9.3.8 Social Impact Assessment 39

10.0 ADVERSE EVENTS 39

10.1 Definition 39
10.2 Assessment of Severity of Adverse Events 40
10.3 Relationship to Investigational Product 40
10.4 Serious Adverse Events 41
10.5 Clinical Management of Adverse Events 42
10.6 Pregnancy 42
10.7 Intercurrent HIV Infection 42
10.8 Serious Event Prior to Investigational Product Administration 42

11.0 MANAGEMENT OF HIV ISSUES DURING AND FOLLOWING STUDY 43

11.1 HIV Testing 43
11.2 Social Discrimination as a Result of an Antibody Response to Vaccine 43
11.3 HIV Infection 43
11.3.1 Counselling 43
11.3.2 Referral for Support, Care and Treatment 44

12. DISCONTINUATION OF VACCINATIONS AND/OR WITHDRAWAL FROM STUDY 44

12.1 Discontinuation of Vaccinations 44
12.2. Follow-Up after Discontinuation of Further Vaccinations 45
12.3 Withdrawal from the Study (Early Termination) 45

13.0 DATA HANDLING 46

13.1 Data Collection and Record Keeping at the Study Site 46
13.2 Data Entry at the Study Site 46
13.3 Data Analysis 46

14.0 STATISTICAL CONSIDERATIONS 47

14.1 Sample Size 47
14.2 Null Hypothesis 47
14.3 Statistical Power and Analysis 47
Table 14.3-1: Vaccine versus Placebo 48
Table 14.3-2: Comparison of 2 vaccine groups 48

15.0 QUALITY CONTROL AND QUALITY ASSURANCE 48

16.0 DATA AND BIOLOGICAL MATERIAL 49

17.0 ADMINISTRATIVE STRUCTURE 49

17.1 Protocol Safety Review Team 49
17.2 Safety Review Board 49
17.2.1 Content of Interim Safety Review 50
17.3 Criteria for Pausing the Study 50
17.4 Study Supervision 51
17.5 Study Monitoring 51
17.6 Investigator's Records 51

18.0 INDEMNITY 51

19.0 PUBLICATION 51

20.0 ETHICAL CONSIDERATIONS 52

APPENDIX A: SCHEDULE OF PROCEDURES GROUPS 1-3 53
APPENDIX B: SCHEDULE OF PROCEDURES GROUPS 4 AND 5 55
APPENDIX C: ADVERSE EVENT SEVERITY ASSESSMENT TABLE 57
References 76

ABBREVIATIONS

Abbreviation	Term
Ad	Adenovirus (serotype)
ADCC	Antibody-Dependent Cell-Mediated Cytotoxicity
ADCVI	Antibody-Dependent Cell-Mediated Virus Inhibition
AE	Adverse Event
AIDS	Acquired Immunodeficiency Syndrome
ALC	Absolute Lymphocyte Count
ALT	Alanine Aminotransferase
ANC	Absolute Neutrophil Count
AOU	Assessment of Informed Consent Understanding
AST	Aspartate Aminotransferase
CL	Confidence Limit
CMI	Cell Mediated Immunity
CMO	Chief Medical Officer
CRC	Clinical Research Centres
CRF	Case Report Form
CTL	Cytotoxic T Lymphocyte
CTA	Clinical Trial Agreement
DCC	Data Coordinating Centre
DNA	Deoxyribonucleic Acid
ELISPOT	Enzyme Linked Immunosorbent Spot Assay
EP	Electroporation
ERB	Ethics Review Board
GCP	Good Clinical Practice
GMP	Good Manufacturing Practice
HBsAg	Hepatitis B Surface Antigen
HCG	Human Chorionic Gonadotropin
HCV	Hepatitis C Virus
HIL	Human Immunology Laboratory
HIV	Human Immunodeficiency Virus
HLA	Human Leukocyte Antigen
IAVI	International AIDS Vaccine Initiative
ICH	International Conference on Harmonisation
ICS	Intracellular Cytokine Staining
IEC	Independent Ethics Committee
IL	Interleukin
IM	Intramuscular
IM/EP	Intramuscular by *in vivo* electroporation
IP	Investigational Product
IRB	Institutional Review Board
IUD	Intrauterine Device
Kg	Kilogram
mg	Milligram
mL	Milliliter
mm	Millimeter

Abbreviation	Term
NHP	Non-human Primate
PBMC	Peripheral Blood Mononuclear Cells
PSRT	Protocol Safety Review Team
Ad35	Adenovirus Serotype 35
SAE	Serious Adverse Event
SHIV	Simian-Human Immunodeficiency Virus
SAP	Statistical Analysis Plan
SIV	Simian Immunodeficiency Virus
SOP	Standard Operating Procedure
SOM	Study Operations Manual
SRB	Safety Review Board
STI	Sexually Transmitted Infection
ULN	Upper Limit of Normal
vp	Viral Particle
VSV	Vesicular Stomatitis Virus

2.0 SIGNATURE PAGE

The signatures below constitute the approval of this protocol and the appendices and provide the necessary assurances that this study will be conducted in compliance with the protocol, Good Clinical Practice (GCP) and the applicable regulatory requirement(s).

Sponsor:

Signed: ______________________________ Date: ____________

Patricia Fast, MD, PhD
Chief Medical Officer, Medical Affairs, IAVI

Principal Investigator:

Signed: ______________________________ Date: ____________

Name (please print): ______________________________

Name of institution (please print): ______________________________

3.0 INTRODUCTION AND BACKGROUND INFORMATION

According to the Joint United Nations Programme on HIV/AIDS and the World Health Organization, as of the end of 2009 approximately 33.3 million people were estimated to be living with HIV/AIDS, with 95% residing in the developing world. It is estimated that in 2009 alone, 2.6 million people were newly infected with HIV, there were approximately 7,120 new infections per day and 1.8 million people died of AIDS[1].

Sub-Saharan Africa remains the region most affected by the AIDS pandemic, accounting for 1.8 million (69%) new infections in adults, a majority of which are women, 92% of new infections in children and 72% of all AIDS deaths recorded in 2009. It is estimated that 1.8 million people were newly infected with HIV in 2009, bringing to 22.5 million the total number of people living with HIV in the region.

There is an urgent need to strengthen and scale-up existing prevention methods such as behavioural interventions, condom use, treatment of sexually transmitted diseases, harm reduction and male circumcision. New prevention strategies to control the epidemic and prevent new infections, including pre-exposure prophylaxis, HIV preventive and therapeutic vaccines and topical microbicides need to be explored and access ensured. The development of an efficacious preventive vaccine against HIV-1 remains the best hope for controlling the HIV/AIDS pandemic[2 3].

Data from a recent community-based efficacy trial in Thailand (RV144) demonstrated a modest 31.2% protection against HIV acquisition. The vaccination regimen consisted of priming with the canarypox vector ALVAC-HIV, expressing HIV *gag, pro*, and *env* genes, and boosting with recombinant HIV Env protein. The regimen did not decrease HIV viral load in vaccine recipients who acquired HIV. Limited cellular immune responses were detected, while binding antibodies were detected in a majority of vaccine recipients, with very limited detectable neutralizing antibody responses to HIV. Studies of the immune correlates of protection are ongoing[4]. These results suggest that more potent vaccine regimens will be required to generate HIV-1 immune responses providing more significant protection[5].

Experimental and natural history studies suggest that both HIV-specific neutralizing/functional antibodies and long-lasting effector and central memory HIV-specific CD8+ and CD4+ T-cell responses are needed in both systemic and mucosal compartments to effectively control HIV infection[6 7 8 9 10 11 12 13].

Neutralizing antibodies against circulating isolates are induced principally by the envelope glycoprotein (Env) of HIV and could potentially confer sterilizing immunity against HIV, as suggested by non-human primate SHIV challenge studies [14 15 16]. However, attempts to design appropriate immunogens have failed. This failure has been a major drawback for env-based HIV vaccines, since current immunogens afford only very narrow protection against HIV strains that are closely related to the vaccine antigen[17 18]. The search for an immunogen able to induce broad cross-protective and long-lasting neutralizing/functional antibodies remains difficult and critical [19 20].

Evidence that CD8+ cytotoxic T lymphocytes (CTL) can control HIV replication in the absence of antibodies has been demonstrated in HIV-infected subjects with a reduction in viremia in acute infection temporally associated with HIV-1-specific CTL[21 22 23]. The role of CTL was further

suggested in the SIV macaque model [24 25 26 27 28].

Issues of quantity, quality and location impact the ability of CD8+ T cells to mediate protection from infection[29]. To protect from viruses that remain within the cells of the host and cause persistent infection, such as HIV, the immune system has evolved CD4+ and CD8+ T cells. Both T cell subtypes are recruited in tissues by dendritic cells sensitized through the Toll-like receptors to the presence of pathogens. CD4+ T cells provide help to naive CD8+ T cells so they can proliferate and acquire the ability to recognize foreign antigens presented on the surface of the infected cells and kill them. Naive CD8+ and CD4+ T cells become memory T cells once they have encountered the antigen. There are two main subtypes, effector and central memory CD8+ T cells, that can be differentiated by their surface expression of receptors, cytokine production and ability to proliferate[30].

Preservation and/or restoration of intestinal CD4+ memory T cells seems to be associated with protection from challenge and control of viremia in the non-human primate SIV challenge model[31]. These results suggest that protection against pathogenic lentiviral infection or disease progression may correlate with preservation of mucosal CD4+ T cells. In animals, accumulating evidence suggests that HIV-specific CD4+ T cells are equally crucial for the induction of a protective immune response against HIV[32]. Furthermore, the analysis of immune responses in HIV-infected individuals suggests a crucial role for CD4+ T cells[33]. CD4+ T cells provide essential help to CD8+ effector T cells in long-term non-progressors[34 35 36]. In contrast, the absence of HIV-specific CD4+ T cells in chronically infected individuals seems to be related to an impairment of CD8+ T cell maturation[37]. Altogether, these data suggest that an effective HIV vaccine that controls viral replication should induce strong HIV-specific CD4+ and CD8+ T cell responses.

A heterologous prime-boost vaccination regimen involves priming the immune system to a target antigen delivered by one vaccine and then selectively boosting the immune response by repeat administration of the antigen by a second and distinct vaccine[38]. The synergistic enhancement of immunity to the target antigen is reflected in an increased number of antigen-specific T cells, selective enrichment of high avidity T cells and increased efficacy against pathogenic challenge[39]. Heterologous HIV immunogens derived from different clades for sequential priming and boosting predominantly stimulated T-cell immunity against conserved epitopes[40], whereas a single vaccine derived from one clade or the mixture of multiple vaccines from different clades primarily raised T-cells against less conserved or non-conserved epitopes[41 42].

This study proposes to test a prime-boost regimen to elicit both HIV-specific CD4+ T cells and CD8+ T cells with a recombinant Clade B multiantigen HIV pDNA (HIV-MAG) vaccine co-administered with IL-12 pDNA (GENEVAX® IL-12) given intramuscularly by electroporation, followed by a recombinant adenovirus serotype 35-based vector (Ad35-GRIN/ENV, Clade A fusion gene *gag, reverse transcriptase, integrase* and *nef and gp140 gene*). In this study, the immune response induced when Ad35GRIN/ENV given as a prime followed by HIV pDNA (HIV-MAG) vaccine co-administered with IL-12 pDNA given intramuscularly by electroporation will also be evaluated.

Ad35-GRIN/ENV was selected as both prime and boost vaccine, as the ongoing Phase 1 clinical study of Ad35-GRIN/ENV (IAVI B001) suggests that the vaccine is generally well-tolerated at the dosage proposed in this study, and that the vaccine is immunogenic after one dose[43].

3.1 Study Rationale

We propose to conduct a Phase 1 randomized, placebo-controlled, double-blind clinical trial in HIV-uninfected healthy adult volunteers at low-risk for HIV infection to evaluate the safety, tolerability and immunogenicity of a fixed dose of the HIV-MAG pDNA encoding *gag-pol*, *nef-tat-vif* and *env* genes, co-administered with two dosage levels of plasmid human IL-12 (IL-12 pDNA) delivered IM/EP, followed by recombinant Ad35-GRIN/ENV HIV vaccine delivered IM. The hypotheses tested for this prime-boost regimen are:

- The proposed prime-boost vaccine regimens will be safe.
- GENEVAX® IL-12 co-administration with HIV-MAG will increase HIV vaccine specific responses.
- The Ad35 GRIN /ENV vaccine will boost HIV-specific CD4+ and CD8+ T-cells in a majority of vaccine recipients.
- HIV-MAG co-administered with GENEVAX® IL-12 will boost HIV-specific immune responses induced by the Ad35-GRIN/ENV vaccine.

Plasmid DNA vaccines have been tested in a variety of investigational clinical settings, and given alone, they have been weakly immunogenic in human trials. Various strategies have been used to improve the immunogenicity of DNA vaccines: i) Electroporation (EP) has been shown to be an efficient means to introduce DNA into cells [44 45 4647]. The Ichor TriGrid™ Delivery System (TDS-IM) is an integrated fully automated administration device. Please refer to the Ichor TDS-IM Instructions for Use in the B004 Study Operation Manual. ii) Co-administration of immune-modulators such as IL-12 pDNA as molecular adjuvants has shown promise in preclinical studies [48 49 50]; however, the initial clinical studies with IL-12 pDNA have been disappointing (HVTN studies 060, 063, 070 in which DNA+IL12 pDNA were given IM by standard needle injection).

Recently the HVTN 080 Phase 1 clinical study, combining both strategies, evaluated the safety and immunogenicity of PENNVAX™-B (plasmid DNA *gag, pol, env*) alone or with GENEVAX®IL-12 administered by IM/EP. In the HVTN 080 study, volunteers received 3 vaccinations of PENNVAX™-B, alone (10 subjects) or in combination with of GENEVAX® IL-12 (30 subjects) at 0, 1, and 3 months. The vaccine (3,000 mcg total dose) and GENEVAX® IL-12 (1,000 mcg) were delivered by IM/EP (Inovio's CELLECTRA® device). Immune responses were measured by intracellular cytokine secretion (ICS) assay using global putative T-cell epitope synthetic HIV peptides. The results of the study indicate that GENEVAX® IL-12 co-administered with PENNVAX™-B and delivered by *in vivo* electroporation was safe and well-tolerated. In addition, GENEVAX® IL-12 increased the proportion of vaccine recipients with CD4+ or CD8+ T-cell responses by 2-fold. Please refer to the DNA+IL-12 Investigator's Brochure and Safety Attachment (Version 2.0, May 2011).

IAVI also recently conducted an SIV challenge study in non-human primates (NHP) with a prime boost regimen, consisting of SIV plasmid DNA vaccine in the presence or absence of IL-12 pDNA, delivered IM by electroporation (TriGrid™ Delivery System [TDS-IM] device by Ichor) and corresponding rAd5 constructs given IM 4 months later. SIV DNA plasmid co-delivered with IL-12 pDNA given by IM/EP induced a greater frequency of polyfunctional T cells, compared to the groups that received the SIV plasmid DNA administered by EP without IL-12 pDNA. Following repeated intra-rectal challenge with a low dose of SIVmac239, a 4-log reduction in SIV set-point virus load was observed in vaccinated macaques compared to naive controls ($p \leq 0.01$); moreover, 5 of 6 animals controlled viremia to very low or undetectable levels. This level of viral control is unprecedented for the DNA prime - rAd5 boost strategy and the near 4-log

reduction in virus load achieved in this NHP model of SIV infection, warrants investigation of a similar heterologous prime-boost regimen in humans. Please refer to the Ad35-GRIN/ENV Investigator's Brochure (Version 4.0, May 2011).

Taken together, the efficacy data from this NHP SIV challenge study and the immunogenicity data from the HVTN 080 Phase 1 clinical study provide a strong rationale to initiate clinical testing of HIV-MAG + GENEVAX® IL-12 prime given IM/EP, followed by an Ad35-GRIN/ENV boost.

3.2 Experience with HIV-MAG

HIV-MAG, the HIV-1 multiantigen pDNA vaccine [51], alone or co-administered with GENEVAX® IL-12, given IM by electroporation or standard needle injection is currently being tested in HIV-infected individuals in the US, but no safety data are available yet (ACTG 5281).

Pre-clinical data in rabbits indicate that the 2 HIV-MAG pDNA constructs, expressing a Gag/Pol fusion protein, a Nef/Tat/Vif fusion protein and Env protein, co-administered with human IL-12 pDNA given either IM or IM/EP are well-tolerated and do not cause any adverse effects. No significant differences in biodistribution or persistence were observed when vaccine pDNA was administered by TDS-IM/EP or by conventional IM injection.

In another study in rabbits, repeated dosing with HIV-MAG with or without GENEVAX® IL-12 given IM/EP as prime followed by two doses of recombinant viral vector boost (Vesicular Stomatitis Virus vaccine [VSV HIV gag]) as boost was also well tolerated.

Preclinical immunogenicity studies in mice and rhesus macaques showed that the HIV-MAG co-administered with IL-12 by IM/EP was able to induce HIV-1 antigen specific cell-mediated immune (CMI) responses as determined by IFN-γ ELISPOT assay. In macaques, each HIV-MAG pDNA construct co-administered with GENEVAX® IL-12 also elicited humoral immune responses measured by ELISA.

Please see the most recent version of the Profectus Biosciences, Inc. (PBS) HIV-MAG – IL12 Investigator's Brochure (Version 2.0, May 2011) for a full description of the preclinical safety profile of this candidate vaccine.

3.3 Experience with recombinant human IL-12 pDNA as molecular adjuvant for HIV pDNA vaccines

No clinical studies have yet been conducted testing HIV-MAG vaccine co-administered with GENEVAX® IL-12.

However, other HIV-1 pDNA vaccines expressing antigens similar to HIV-MAG have been tested in Phase 1 clinical studies in combination with GENEVAX® IL-12. The NIH-sponsored clinical trials (HVTN 060, 063, 070) in which different candidate HIV-1 pDNA vaccines co-administered with the GENEVAX® IL-12 given intramuscularly (IM) by needle injection were tested in approximately 250 healthy HIV-uninfected adults. No major safety concerns have been noted in these studies.

In HVTN 080, an HIV-1 pDNA vaccine (PENNVAX™-B gag, pol, env) mixed with GENEVAX® IL-12 was administered IM by *in vivo* electroporation (EP) using Inovio's Cellectra device. Final safety data are not yet available, but no major safety concerns have been reported. Preliminary

immunogenicity data from HVTN 080 indicate that IL-12 increases the frequency of immune responses to the HIV-1 proteins as measured by ICS (CD4+: 81%, CD8+: 52% with IL-12 versus CD4+: 44%, CD8+: 33% without IL-12) 2 weeks post 3rd administration of PENNVAX™-B and GENEVAX® IL-12.

Please see the Profectus Biosciences Inc. (PBS) HIV-MAG – IL12 Investigator Brochure (Version 2.0, May 2011) for a full description of the preclinical and/or clinical safety profile of the HIV-MAG vaccine and the IL-12 pDNA as molecular adjuvant.

3.4 Experience with Ichor TriGrid™Delivery System for *in vivo* electroporation

To date, the TDS-IM has been utilized as the means of DNA vaccine administration in two completed clinical trials and is currently being evaluated in three ongoing clinical studies. These studies include completed studies of a xenogeneic tyrosinase DNA vaccine candidate in patients with Stage IIB-IV melanoma and a multigenic HIV-1 DNA vaccine candidate (ADVAX) in healthy, HIV uninfected volunteers (IAVI C004). The currently ongoing clinical studies include testing of a multi-epitope malaria DNA vaccine in healthy volunteers, assessment of an epitope based TRP-2 melanoma vaccine in patients with AJCC stage III-IV melanoma, and a multi-antigen HIV DNA vaccine administered with or without a DNA based human IL-12 adjuvant in HIV infected individuals. To date, the five trials have enrolled over 90 subjects in electroporation arms of the studies (including subjects receiving either the DNA vaccine candidate or placebo). The device has been used for administration at DNA doses of up to 4.0 mg. Subjects have been administered the vaccine either as a single injection in one muscle site or as two injections in two separate muscle sites. To date, subjects administered the DNA dose as a single injection have received up to five administrations (i.e., five total TDS-IM injections) while subjects administered the DNA dose in two injections have received up to four administrations (i.e., eight total TDS-IM injections).

Adverse events reported in association with use of the device include discomfort/pain during procedure application, minor cutaneous bleeding at the site of injection, and transient injection site soreness of mild to moderate severity, typically resolving within 24-72 hours following administration. Several subjects in the two melanoma studies have reported lightheadedness immediately following procedure application which, in some cases, was accompanied by a decrease in blood pressure. One subject, enrolled in the xenogeneic tyrosinase study, experienced a brief syncopic episode (~30 seconds duration) shortly after procedure application. The subject recovered without incident. At the time of enrollment, the subject indicated a life long history of sinus bradycardia of unknown origin, which was confirmed by electrocardiogram during screening. Multiple electrocardiograms performed after the syncopic episode indicated no changes from pre-procedure baseline. Based on the judgment of the investigator, the subject was withdrawn from the study and the study eligibility criteria modified to exclude subjects with sinus bradycardia. No other serious or unanticipated adverse events attributed to the device or administration procedure have been observed during the five studies.

The results of the completed HIV-1 ADVAX vaccine study in healthy volunteers have been published.[52] Briefly, results from this study indicate that EP based delivery with the TDS-IM device at ADVAX DNA doses ranging from 0.2 – 4.0 mg was safe and effective in improving the magnitude, breadth and durability of cellular immune responses to a DNA vaccine candidate. Assessment of the tolerability of the EP procedure by questionnaire after each administration indicates that the procedure is acceptable for healthy, HIV-uninfected volunteers.

3.5 Experience with Ad35-GRIN/ENV

Ad35-GRIN/ENV consists of two co-formulated Ad35 vectors:

- Ad35-GRIN is a replication-incompetent, recombinant Adenovirus serotype 35 expressing HIV-1 Clade A genes (*gag, reverse transcriptase, integrase* and *nef* (GRIN).
- Ad35-ENV is a replication-incompetent, recombinant Adenovirus serotype 35 expressing HIV-1 clade A gp 140 gene (ENV).

A Phase 1 placebo-controlled, double-blinded, randomized dose-escalation trial (IAVI B001) to evaluate the safety and immunogenicity of Ad35-GRIN/ENV HIV vaccine at three dosage levels (Group A: 2×10^9 vp, Group B: 2×10^{10} vp, and Group C: 2×10^{11} vp) and Group D: Ad35-GRIN vaccine at the dosage of 1×10^{10} vp is being conducted. Vaccine is administered intramuscularly at months 0 and 6. Each of the four groups of 14 Ad35 seronegative volunteers includes 10 vaccine and 4 placebo recipients for a total of 56 volunteers.

No related serious adverse event has been reported to date. Reactogenicity events reported seem dose-dependent. Local reactogenicity events are mostly pain and tenderness at the site of injection, mostly mild or moderate in Group A, B, and D volunteers and mild to severe in Group C volunteers. Systemic reactogenicity events reported included chills, fever, malaise, headache, myalgia, and arthralgia. These reactions were predominantly mild or moderate, except in Group C, in which more severe events were observed. All reactogenicity events were transient and resolved spontaneously[53].

In total, 165 non-serious adverse events have been reported (as of May 5, 2011). The frequency was not dose-related. Two events were assessed as severe (Grade 3): deep vein thrombosis and anxiety disorder; both were considered unrelated to the vaccine. Fifty-eight (58) events were assessed as moderate (Grade 2) in severity: only one was considered possibly related to vaccine (influenza-like illness). One hundred and five (105) events were assessed as mild (Grade 1) in severity. Nine events were considered as possibly related to vaccine (diarrhea, injection site haemorrhage, influenza-like illness, upper respiratory tract injection, 2 nasal congestions, pharyngo-laryngeal pain, pharyngitis, and naso-pharyngitis), one as probably related (injection site anesthesia) and one definitely related (injection site swelling). All other events were considered unrelated or unlikely related to vaccine.

Moderate or greater abnormal clinical laboratory values were observed in five volunteers: elevated AST in two subjects, low haemoglobin in two women (all moderate and not considered as related to vaccination), and elevated ALT in one subject (severe and not considered as related to vaccination).

Two other Phase 1 double-blinded, placebo-controlled, randomized trials in HIV-uninfected, healthy adult volunteers are currently ongoing testing the two Ad35 HIV-1 vaccine vectors separately each:

i) IAVI B002 at four Clinical Research Centres (CRCs) evaluating the safety and immunogenicity of Ad35-GRIN given as either as prime or as boost or co-administered with an adjuvanted HIV protein vaccine;

ii) IAVI B003 at seven CRCs testing Ad35-EnvA in homologous and heterologous prime-boost combinations with Ad26ENVA.01. No data are available yet.

Please see the most recent version of the Ad35-GRIN/ENV Investigator's Brochure (Version 4.0, 29 June 2011) for a full description of the preclinical and clinical safety profile of this candidate vaccine.

4.0 STUDY OBJECTIVES

4.1 Primary Objectives

1. To evaluate the safety and tolerability of HIV-MAG with or without co-administered GENEVAX® IL-12 given intramuscularly by *in vivo* electroporation (IM/EP) using the Ichor Medical Systems TriGrid™ Delivery System (TDS-IM), followed by Ad35-GRIN/ENV in each of four different regimens

2. To evaluate the safety and tolerability of Ad35-GRIN/ENV followed by HIV-MAG with co-administered GENEVAX® IL-12 given intramuscularly by *in vivo* electroporation (IM/EP) using the Ichor Medical Systems TriGrid™ Delivery System (TDS-IM)

4.2 Secondary Objectives

1. To determine whether the HIV-specific immune responses induced by HIV-MAG are increased by co-administration of GENEVAX® IL-12 in a prime-boost regimen with Ad35-GRIN/ENV given as boost and if so, which dosage level induces the higher immune responses

2. To compare HIV-specific immune responses induced by HIV-MAG co-administered with GENEVAX® IL-12 IM/EP once at M0 followed by Ad35-GRIN/ENV IM at M4 with those induced by HIV-MAG co-administered with GENEVAX® IL-12 IM/EP trice at M0,1,2 followed by Ad35-GRIN/ENV IM at M6

3. To compare HIV-specific immune responses induced by Ad35-GRIN/ENV IM prime at M0 followed by HIV-MAG co-administered with GENEVAX® IL-12 IM/EP boost at M4 with those induced by HIV-MAG co-administered with GENEVAX® IL-12 IM/EP prime at M0 followed by Ad35-GRIN/ENV IM boost at M4

5.0 STUDY ENDPOINTS AND STUDY DESIGN

5.1 Study Endpoints

5.1.1 Primary Endpoints

Safety and tolerability:
To assess safety and tolerability of the different prime-boost regimens:

1. Proportion of volunteers with moderate or greater reactogenicity (i.e., solicited adverse events) during a 7 day follow-up period after each vaccination

2. Proportion of volunteers with moderate or greater and/or vaccine-related unsolicited adverse events (AEs) including safety laboratory (biochemical, haematological) parameters, from the day of each vaccination up to 28 days post each vaccination.

3. Proportion of volunteers with vaccine related serious adverse events (SAEs) collected throughout the study period

5.1.2 Secondary Endpoints

Immunogenicity:
To assess (qualitative and quantitative) immune responses elicited by the different prime-boost regimens:

1. Proportion of volunteers with HIV-1 specific T-cell responses quantified by IFN-γ ELISPOT and magnitude of responses

2. Frequency and magnitude of polyfunctional HIV-1 specific CD4+ or CD8+ T-cell responses assessed by Intracellular Cytokine Staining and Polychromatic Flow Cytometry (ICS/PFC) measuring a panel of cytokines, functional and phenotypic markers after stimulation with HIV-1 antigen peptide pools

3. Epitope mapping to determine specificity and breadth of the responses

5.1.3 Exploratory Endpoints

Immunogenicity:
Additional immunogenicity assessments may include:

1. Binding antibodies to HIV antigens (frequency and magnitude)

2. Neutralizing antibodies to HIV antigens (seropositivity rates and magnitude of antibody titers)

3. Neutralizing antibodies to the Ad35 vector (frequency and magnitude)

Responses may be further characterized to include:

1. Ability of CD8 cells to inhibit HIV replication in a Viral Inhibition Assay (VIA)

2. Capacity of antigen specific T cells to proliferate

3. Flow cytometry to assess markers for memory, exhaustion and activation, and secretion of cytokines other than IFN-γ, IL-2 and TNF-α

4. Repertoire analysis to assess B cell response

Serum antibodies against human IL-12:

1. Proportion of volunteers/vaccine recipients who develop antibodies against human IL-12

EP Tolerability:

1. Proportion of volunteers judging the procedure as acceptable

5.2 Study Design

The study is a randomized, double-blind placebo-controlled trial.

Table 5.2-1: Study Design

		Months 0, 1, 2	Month 6
Study Groups	N vaccine / placebo	Prime Vaccine (dosage, delivery)	Boost Vaccine (dosage, delivery)
1	**12/3**	HIV-MAG (3,000mcg) (IM/EP*)	Ad35-GRIN/ENV ($2x10^{10}$vp*** IM**)
2	**12/3**	HIV-MAG (3,000mcg) + GENEVAX® IL-12 (100mcg)(IM/EP)	Ad35-GRIN/ENV ($2x10^{10}$vp, IM)
3	**12/3**	HIV-MAG (3,000mcg) + GENEVAX® IL-12 (1000mcg)(IM/EP)	Ad35-GRIN/ENV ($2x10^{10}$ vp, IM)
		**Month 0**	**Month 4**
4	**12/3**	HIV-MAG (3,000mcg) + GENEVAX® IL-12 (1000mcg)(IM/EP)	Ad35-GRIN/ENV ($2x10^{10}$ vp, IM)
5	**12/3**	Ad35-GRIN/ENV ($2x10^{10}$ vp, IM)	HIV-MAG (3,000mcg) + GENEVAX® IL-12 (1000mcg) (IM/EP)
**Total**	**75 (60/15)**		

*IM/EP=intramuscular by *in vivo* electroporation
**IM=intramuscularly
*** vp=viral particles

5.2.1 Duration of the Study

Volunteers will be screened up to 42 days before vaccination and will be followed for 12 months after the initial vaccination. It will take approximately 3 months to enrol 75 volunteers. The anticipated study duration for each volunteer is approximately 13 months from screening through last study visit.

5.2.2 Study Population

The study population consists of healthy male or female adults aged 18-50 years at low risk for HIV infection, who are willing to undergo HIV testing, use an effective method of contraception, and who in the opinion of the investigator or designee, understand the study and provide written informed consent.

Approximately 75 volunteers (60 vaccine recipients, 15 placebo recipients) who meet all eligibility criteria will be included in the study. An over-enrolment of up to 10% (up to 83 volunteers total) will be permitted in the study to facilitate rapid enrolment.

5.2.3 Inclusion Criteria

1. Healthy male or female, as assessed by a medical history, physical exam, and laboratory tests;
2. At least 18 years of age on the day of screening and has not reached his/her 51st birthday on the day of first vaccination;
3. Willing to comply with the requirements of the protocol and available for follow-up for the planned duration of the study;
4. In the opinion of the Principal Investigator or designee, and based on Assessment of Informed Consent Understanding (AOU) results, has understood the information provided and potential risks linked to vaccination and participation in the trial; written informed consent will be provided by the volunteer before any study-related procedures are performed;
5. Willing to undergo HIV testing, risk reduction counselling, receive HIV test results and committed to maintaining low risk behaviour for the trial duration;
6. If a female of childbearing potential, willing to use an effective non-barrier method of contraception (hormonal contraceptive or intrauterine device [IUD]) from screening until at least 4 months after the last study vaccination;
7. Assessed by the clinic staff as being at “low risk” for HIV infection on the basis of self-reported sexual behaviour within the 12 months prior to enrolment defined as follows:
    a. Sexually abstinent, or
    b. Had two or fewer mutually monogamous relationships with partners who did not use illicit drugs, or
    c. Had two or fewer partners believed to be HIV-uninfected and who did not use illicit drugs (illicit drug use or abuse that includes any injection drugs, methamphetamines [crystal meth], heroin, cocaine, including crack cocaine or chronic marijuana abuse) and with whom he/she regularly used condoms for vaginal and anal intercourse;
8. All female volunteers must be willing to undergo urine pregnancy tests at time points indicated in the Schedule of Procedures (Appendices A, B) and must test negative prior to each study vaccination;
9. All sexually active males (unless anatomically sterile or in a monogamous relationship with a female partner who uses a documented non-barrier method of birth control) must be willing to use an effective method of contraception (such as consistent condom use) from the day of first vaccination until at least 4 months after the last vaccination;
10. Willing to forgo donations of blood or any other tissues during the study and, for those who test HIV-positive due to trial vaccination (vaccine-induced HIV seropositivity), until the anti-HIV antibody titres become undetectable.

5.2.4 Exclusion Criteria

1. Confirmed HIV-1 or HIV-2 infection;
2. Any clinically relevant abnormality on history or examination including history of immunodeficiency or autoimmune disease; use of systemic corticosteroids (the use of topical or inhaled steroids is permitted); immunosuppressive, anti-cancer, anti-tuberculosis or other medications considered significant by the investigator within the previous 6 months;
3. Any clinically significant acute or chronic medical condition that is considered progressive, or in the opinion of the investigator, makes the volunteer unsuitable

for participation in the study;

4. Reported risky behaviour for HIV infection within 12 months prior to vaccination, as defined by:
    - Unprotected sexual intercourse with a known HIV-infected person, a partner known to be at high risk of HIV infection or a casual partner (i.e., no continuing established relationship)
    - Engaged in sex work
    - Frequent excessive daily alcohol use or frequent binge drinking or chronic marijuana or any other use of use of illicit drugs
    - History of newly-acquired syphilis, gonorrhoea, non-gonococcal urethritis, HSV-2, chlamydia, pelvic inflammatory disease (PID), trichomonas, muco-purulent cervicitis, epididymitis, proctitis, lymphogranuloma venereum, chancroid, or hepatitis B;
    - Three or more sexual partners
5. If female, pregnant or planning a pregnancy within 4 months after last study vaccination; or lactating;
6. Asthma requiring high-dose oral or inhaled corticosteroids;
7. Bleeding disorder that was diagnosed by a physician (e.g., factor deficiency, coagulopathy or platelet disorder that requires special precautions) (Note: A volunteer who states that he or she has easy bruising or bleeding, but does not have a formal diagnosis and has IM injections and blood draws without any adverse experience, is eligible);
8. History of splenectomy;
9. Any of the following abnormal laboratory parameters listed below:
    - Haematology
        - Haemoglobin ≤9.0 g/dL **or** ≤5.55 mmol/L
        - Absolute Neutrophil Count (ANC): ≤ 1000/mm^3 **or** ≤1.0 x 10^9/L
        - Absolute Lymphocyte Count (ALC): ≤ 500/mm^3 **or** ≤ 0.5 x 10^9/L
        - Platelets: ≤ 90,000 ≥ 550,000/ mm^3 **or** ≤ 90 x 10^9 ≥ 550 x 10^9/L
    - Chemistry
        - Creatinine: >1.4 x upper limit of normal (ULN)
        - AST: ≥2.6 x ULN
        - ALT: ≥2.6 x ULN
    - Urinalysis: clinically significant abnormal dipstick confirmed by microscopy
        - Protein 2+ or more
        - Blood 2+ or more (not due to menses);
10. Receipt of live-attenuated vaccine within the previous 60 days or planned receipt within 60 days after vaccination with Investigational Product; or receipt of other vaccine, allergy treatment with antigen injections or tuberculin skin test within the previous 14 days or planned receipt within 14 days after vaccination with Investigational Product;
11. Receipt of blood transfusion or blood-derived products within the previous 3 months;
12. Participation in another clinical trial of an Investigational Product currently, within the previous 3 months or expected participation during this study;
13. Prior receipt of another investigational HIV vaccine candidate (Note: receipt of

placebo in a previous HIV vaccine trial will not exclude a volunteer from participation if documentation is available and the Medical Monitor gives approval);

14. History of severe local or systemic reactogenicity to vaccines (e.g., anaphylaxis, respiratory difficulty, angioedema);
15. Positive for Hepatitis B surface antigen (HbsAg), positive for antibodies to Hepatitis C virus (HCV) or active syphilis;
16. Psychiatric condition that precludes compliance with the protocol. Specifically excluded are persons with psychoses within the past 3 years, ongoing risk for suicide, or history of suicide attempt or gesture within the past 3 years.
17. History of allergy or hypersensitivity to bupivacaine or any amide-type (local) anesthetics, such as lidocaine (Xylocaine®), mepivacaine (Polocaine®/ Carbocaine®), etidocaine (Duranest®), bupivacaine (Marcaine®), or prilocaine or known hypersensitivity to any component of the products tested
18. History of allergy or hypersensitivity to latex
19. Chronic skin problems such as eczema or psoriasis
20. Presence of an implantable device [e.g. Automated Implantable Cardioverter-Defibrillator (AICD), pacemaker, {pain} medication administration pumps]
21. Current use of any electronic stimulation device, such as cardiac demand pacemakers, automatic implantable cardiac defibrillator, nerve stimulators, or deep brain stimulators.
22. History of, or known active cardiac disease or a heart condition under the care of a doctor, including cardiac arrhythmia [e.g., supraventricular tachycardia, atrial fibrillation, frequent ectopy, or sinus bradycardia (i.e., <50 beats per minute on exam)] prior to study entry. <u>Note</u>: Slight physiological variation of normal resting heart rate (60 - 100 beats/minute) with respiration is NOT excluded.
23. History of syncope or fainting episode within 1 year of study entry
24. Seizure disorder or any history of prior seizure
25. Skin and subcutaneous tissue thickness > 40 mm as assessed by skin pinch test in either deltoid region
26. If, in the opinion of the Principal Investigator, it is not in the best interest of the volunteer to participate in the trial

5.2.5 Recruitment of Volunteers

Healthy adult male and female volunteers may be recruited through information presented in community organizations, hospitals, colleges, other institutions and/or advertisements to the general public. This information will contain contact details.

6.0 STUDY VISITS

6.1 Screening Period

During Screening, study staff will perform the following procedures:

- Provide and/or review the Informed Consent Document and answer any questions about the study prior to obtaining written informed consent. Complete the Assessment of Understanding (AOU) of Informed Consent which is found in the Study Operations Manual (SOM).

If the volunteer agrees to participate, provides written informed consent and passes the AOU, study staff will:

- Conduct screening assessment
- Conduct HIV test counselling and HIV risk reduction counselling
- Conduct family planning counselling, refer for contraceptive counselling, if necessary, as per site-specific procedures and ensure compliance with respective contraceptive method
- Perform a complete medical history
- Collect concomitant medication information
- Perform a general physical examination (Refer to Section 7.2)
- Perform a measurement of skin fold thickness (Skin Pinch Test)
- Collect specimens for all tests as indicated in the Schedule of Procedures (Appendices A-B)

Screening laboratory test(s) may be repeated once at the discretion of the Principal Investigator or designee to investigate any isolated abnormalities.

If the screening visit occurs more than 42 days prior to the date of 1st vaccination, all screening procedures must be repeated. The complete medical history may be replaced by an interim medical history and the Informed Consent form/ Volunteer Information Sheet should be reviewed.

If a volunteer has signed the consent form, but does not meet the eligibility criteria, the records must be kept at the site.

6.2 Vaccination Visits

Prior to the first vaccination, study staff will:

- Answer any questions about the study
- Review the Informed Consent Document
- Review screening safety laboratory data
- Administer HIV risk assessment
- Conduct family planning counselling as per site-specific procedures and ensure compliance with respective contraceptive method
- Review interim medical history
- Collect concomitant medication information
- Perform a symptom-directed physical examination (Refer to Section 7.2)
- Baseline assessment of local and systemic signs and symptoms (this includes an examination of vaccination site)
- Collect specimens for all tests as indicated in the Schedule of Procedures (Appendices A-B). Obtain pregnancy test results prior to vaccination.

The volunteer will be assigned an allocation number according to the instructions specified in the Study Operations Manual (SOM).

The Investigational Product will be administered as specified in Section 8.4, Administration of Investigational Product and according to the instructions specified in the SOM.

Study staff will observe volunteers closely for at least 30 minutes after each vaccination for any acute reactogenicity. At the end of the observation period study staff will:

- Record vital signs (pulse, respiratory rate, blood pressure and temperature)
- Assess any local and systemic reactogenicity
- Assess any other adverse events
- Ask volunteer to complete EP tolerability assessment (unless volunteer is in Group 5, in which case they will complete the EP tolerability assessment following their Month 4 vaccination only)

Subsequent Vaccination Visits:
Study staff will perform the same procedures as above with the following exceptions:

- Review the routine safety laboratory parameters (Section 9.1.6), as appropriate, from the previous visit prior to each vaccination. If a volunteer has an abnormal laboratory value that is known at the time of vaccination, follow the specified guidelines (Section 12.0)
- Conduct pre HIV-test counselling if an HIV test is scheduled (see Appendices A-B) or clinically indicated
- Provide post-test counselling if the results of a prior HIV test are being communicated to the volunteer
- Do not conduct EP tolerability assessment following vaccinations that do not use EP

6.3 Post-Vaccination Visits

The volunteer will be asked to maintain a Memory Aid from the day of each vaccination and for the next 7 days. Study staff will review the Memory Aid with the volunteer, determine the severity of the reactions through volunteer discussion and record the information on applicable source documentation. There will be scheduled clinic visits 3, 7 and 14 days after each vaccination for an assessment by clinic staff.

The following procedures will be conducted at these visits:

- Collect concomitant medication information
- If any signs or symptoms are present, perform a symptom-directed physical examination
- Assess any adverse events, including local and systemic reactogenicity
- Collect specimens for all tests as indicated in the Schedule of Procedures (Appendices A-B)

6.4 Additional Follow-up Visits

Assessments and procedures will be performed according to the Schedule of Procedures (Appendices A-B).

6.5 Unscheduled Visits

Unscheduled Visits/Contacts are visits/contacts that are not described in the Schedule of Procedures (Appendices A-B). Unscheduled visits may occur any time during the study:

- For administrative reasons, e.g., the volunteer may have questions for study staff or may need to re-schedule a follow-up visit
- To obtain laboratory test results from a previous visit
- For other reasons as requested by the volunteer or site investigator

All unscheduled visits will be documented in the volunteer's study records and on applicable source documents and entered into the study database.

6.6 Final Study Visit or Early Termination Visit

Assessments and procedures will be performed according to the Schedule of Procedures (Appendices A-B).

7.0 STUDY PROCEDURES

7.1 Informed Consent Process

A sample Informed Consent Document consisting of a Volunteer Information Sheet and a Consent Form is provided by the Sponsor to the CRC. This document is made site-specific and translated (if necessary), submitted and approved by the Independent Ethics Committee (IEC)/ Ethics Review Board (ERB).

<u>Volunteer Information Sheet/ Informed Consent Document</u>
A qualified member of the study staff will conduct the informed consent process by reviewing the Volunteer Information Sheet.

The following study-specific elements are included:

1. The vaccines tested in this study cannot cause HIV or AIDS.

2. If a vaccine recipient is exposed to HIV through exposure in the community and acquires HIV, it is not known whether the study vaccine(s) could increase, decrease or have no effect on: a) the risk of becoming infected with HIV; b) if infected, the course of HIV infection and; c) if infected, the time it takes to develop AIDS after being infected

3. The vaccine recipient may develop antibodies against HIV following vaccination, which is desirable but may produce a positive result in a routine HIV antibody test, and that provisions have been made to distinguish between response to vaccine and natural HIV infection during and after the study. In case the volunteer has a positive result due to vaccine-induced antibodies in a routine HIV antibody test, he/she will be followed until the result is no longer positive.

4. Women of childbearing potential should use a reliable non-barrier form of contraception from screening, during the vaccination period and until 4 months after the last vaccination

5. Placebo will be administered in this study and volunteer may receive placebo throughout the study

<u>Consent Form</u>

All volunteers will give their written informed consent to participate in the study on the basis of appropriate information and with adequate time to consider this information and

ask questions. To confirm that the volunteer has understood the information contained in the Volunteer Information Sheet, an Assessment of Informed Consent Understanding (AOU) will be administered, as per site-specific procedures (see SOM).

The volunteer's consent to participate must be obtained by him/her signing (or if illiterate, marking) and dating the Informed Consent Form. The person obtaining consent will also sign. If the volunteer is functionally illiterate, the complete Informed Consent Document (which includes the Volunteer Information Sheet) must be read to him/her in the language that he/she best understands in the presence of an independent literate observer not affiliated with the study, who will sign and date the consent form as an impartial witness.

The signed/marked and dated Informed Consent Document must remain at the study site. A copy of the signed/marked and dated Informed Consent Document will be offered to the volunteer to take home. Those volunteers who do not wish to take a copy will be required to document that they declined to do so.

Family members, sexual partner(s) or spouse(s) will be offered education and counselling regarding a volunteer’s participation in the study ONLY if the participating volunteer has specifically given written consent for such education and counselling.

7.2 Medical History and Physical Examination

At screening, a comprehensive medical history will be collected, including details of any previous vaccinations and reaction to vaccinations, history of STIs and contraceptive practices. At subsequent visits, an interim medical history will be performed.

A general physical examination includes height, weight, examination of skin, respiratory, cardiovascular and abdominal systems, an assessment of cervical and axillary lymph nodes and recording of vital signs (pulse, respiratory rate, blood pressure and temperature). Skin fold thickness (Skin Pinch Test) will be measured at the first physical exam.

A symptom-directed physical examination includes assessment of cervical and axillary lymph nodes, recording of vital signs (pulse, respiratory rate, blood pressure and temperature) and any further examination indicated by history or observation.

7.3 HIV Testing and HIV-test Counselling

Additionally, study staff will perform pre-HIV test counselling (prior to collecting blood for an HIV test) and post-HIV test counselling (when HIV test results are available) according to the Schedule of Procedures (Appendices A-B). For more information on HIV testing and HIV-test counselling, see Section 11.0.

7.4 HIV Risk Reduction Counselling

Study staff will provide HIV risk reduction counselling based on reported individual risk and provide free condoms, as appropriate, at every visit. The procedures for risk reduction counselling will be detailed in site-specific SOPs.

7.5 Family Planning Counselling

Study staff will counsel volunteers about the importance of preventing pregnancies and use of condoms, as well as other effective family planning methods, as appropriate. Free condoms are provided and volunteers may be referred for family planning services either on-site or to a family planning clinic, as necessary and according to site-specific SOPs. Contraceptive methods chosen and compliance will be documented.

7.6 Specimens

Up to approximately 103 mL of blood will be collected at visits, usually from the antecubital fossa, according to the Schedule of Procedures (Appendices A-B).

All specimens will be handled according to the procedures specified in the Study Operations Manual (SOM) and Laboratory Analytical Plan (AP).

In the event of an abnormal laboratory value, volunteers may be asked to have an additional sample collected at the discretion of the Principal Investigator or designee.

7.7 Reimbursement

Volunteers will be reimbursed for their time, effort and for costs to cover their travel expenses to the study site and any inconvenience caused due to study participation. Reimbursement will be made after the completion of each study visit. Site-specific reimbursement amounts will be documented in the site-specific Volunteer Information Sheet and approved by the Ethics Committee.

7.8 Randomization and Blinding

Volunteers will be identified by a unique volunteer identification number.

Volunteers will be randomized across all groups. The randomization schedule will be prepared by the statisticians at the Data Coordinating Centre (DCC) prior to the start of the study. Volunteers will be automatically assigned a specific allocation number as they are enrolled into the data entry system. An unblinding list will be provided to the unblinded site pharmacist by the DCC.

Study staff and volunteers will be blinded to vaccine versus placebo and dosage levels of GENEVAX® IL-12, but not to schedule, number of vaccinations administered and delivery method.

A volunteer will be considered enrolled once she/he has been randomly allocated to a specific vaccination regimen.

Enrolment will be staggered with 1 volunteer across all CRCs enrolled per day for the first 4 volunteers.

Volunteers will be informed about their assignment (vaccine/placebo) at study completion, once the database is locked. Should a study volunteer be unblinded during the study, further administration of the Investigational Product (vaccine or placebo) will be discontinued. The study volunteer will be followed up until the end of the study according to Schedule of Procedures (Appendices A-B).

7.9 Unblinding Procedure for Individual Volunteers

Unblinding of an individual volunteer may be indicated in the event of a medical emergency if the clinical management of the volunteer would be altered by knowledge of the treatment assignment.

The unblinding information should be restricted to a small group of individuals involved in clinical management of the volunteer (e.g., treating physician) and maintain the blind for those responsible for the study assessments.

The reasons for unblinding should be documented and the IAVI Chief Medical Officer, the Medical Monitor and the DCC should be notified as soon as possible. The procedures and contact numbers for unblinding are outlined in the Study Operations Manual (SOM).

7.10 Referral to Long Term Follow-Up Study

To assess the long-term safety of the Investigational Products, study volunteers will, after completion of all visits in this study, be offered participation in a long-term follow-up study for approximately 5 years following the last study injection. This study includes a health assessment questionnaire and HIV testing. Additional blood samples may be collected to assess the persistence of the immune responses in vaccine recipients. A separate informed consent will be administered for this long-term follow-up study.

8.0 INVESTIGATIONAL PRODUCT

8.1 Description

- The HIV-MAG vaccine is manufactured by Boehringer Ingelheim (Austria) and supplied by Profectus Biosciences, Inc.
- GENEVAX® IL-12 is manufactured by Cardinal Health (US) and supplied by Profectus Biosciences, Inc.
- The Ad35-GRIN/ENV vaccine is manufactured by Transgene (France) and supplied by IAVI.
- Sodium Chloride Injection, USP 0.9% will be used as placebo for the HIV-MAG and GENEVAX® IL-12 and Ad35 vaccines.
- The TDS-IM electroporation device is manufactured by California MedTech (US) and Life Science Outsourcing (US) and supplied by Ichor Medical Systems, Inc.

8.1.1 HIV-MAG

The HIV-MAG vaccine consists of two DNA plasmids:

- ProfectusVax HIV-1 subtype B *gag/pol* DNA plasmid
- ProfectusVax HIV-1 subtype B *nef/tat/vif, env* (subtype B primary isolate Env gp160) DNA plasmid

The individual vaccine plasmids are supplied at 3.0 mg/mL in 30 mM citrate buffer pH 6.5 containing 0.15 M NaCl, 0.01% EDTA, and 0.25% bupivacaine-HCl in a 2 mL container with a Daikyo/West Plug stopper and aluminium flip seal. The filling volume per container is 0.8 mL±0.04 mL.

8.1.2 Ad35-GRIN/ENV

Ad35-GRIN/ENV consists of two vectors Ad35-GRIN and Ad35-ENV formulated in a 1:1 ratio and filled into single use vials for intramuscular injection.

- Ad35-GRIN is a recombinant replication-incompetent Adenovirus serotype 35 expressing HIV-1 subtype A *gag*, *reverse transcriptase, integrase*, and *nef* genes.
- Ad35-ENV is a recombinant replication-incompetent Adenovirus serotype 35 expressing HIV-1 subtype A gp140 *env* gene
- Where appropriate, mutations have been introduced to abrogate the normal functions of the HIV antigens.

Ad35-GRIN/ENV is supplied as a frozen sterile formulation in a 4-mL vial with a butyl stopper and aluminum seal. Each vial contains 0.725 mL of vaccine. The volume of administration is 0.5 mL, which will deliver a final dosage of $2x10^{10}$ vp per dose. The dose of the vaccine is provided as a total virus particle count measured by HPLC and expressed as viral particle (vp). The vaccine is formulated in buffer composed of Tris 10 mM pH 8.5, Sucrose 342,3 g/L, 1mM $MgCl_2$, Tween80 54 mg/L and 150 mM NaCl in water for injection (used for diluting the purified bulk). Vaccine is a whitish liquid and limpid or slightly turbid liquid, depending on the virus concentration.

8.1.3. GENEVAX® IL-12

GENEVAX® IL-12 will be provided at 2.0 mg/mL and is formulated in 30 mM citrate buffer (pH 6.5) containing 150 mM NaCl, 0.01% EDTA, and 0.25% bupivacaine-HCl in a 2mL container with Daikyo/West Plug Stopper and aluminium flip seal. The volume per container is 0.9 mL ± 0.04 mL.

8.1.4. Placebo

Commercially available Sodium Chloride Injection, USP 0.9% will be used as placebo.

The summary of the Investigational Products is shown in Table 8.1.4-1.

Table 8.1.4-1: Formulation of Investigational Products

Vaccine/ Placebo	Dosage Level(s)	Total Injected Volume (per site)	Route of Administration
HIV-MAG (alone)	3,000 mcg	1.0 mL (2x0.5 mL)	IM/EP
HIV-MAG+ GENEVAX® IL-12	HIV-MAG (3000 mcg) + GENEVAX® IL-12 (100 mcg)	1.2 mL (2x0.6 mL)	IM/EP
	HIV-MAG (3000 mcg) + GENEVAX® IL-12 (1000 mcg)	1.5 mL (2x0.75mL)	
Ad35-GRIN/ENV	$2x10^{10}$ vp	0.5 mL	IM
Placebo	N/A	1.0 mL (2x0.5 mL)<br>1.2 mL (2x0.6 mL)<br>1.5 mL (2x0.75 mL)	IM/EP
Placebo	N/A	0.5 mL	IM

8.1.5. Ichor Medical Systems TriGrid™ Delivery System (TDS-IM)

The TDS-IM is an electroporation based delivery device designed for IM administration of DNA. Specifically, the device is designed to propagate EP-inducing electrical fields at the site of administration in the presence of the DNA to be delivered. It consists of three components which include a single use Application Cartridge, an Integrated Applicator, and a Pulse Stimulator. The device is a designated Class II medical device for investigational use.

8.1.5.1 Application Cartridge

The Application Cartridge is used to house the agent to be delivered (in a standard syringe) and the electrodes used for EP application. Each Application Cartridge is packaged sterile for single use and is the only subject contact component of the system. It is composed of a plastic, injection molded body that encloses four electrodes arranged to form two equilateral triangles with an adjoining base.

Prior to the administration procedure, the Application Cartridge is removed from the sterile pouch. The agent to be administered is loaded into a standard Becton Dickenson 3.0 cc syringe (BD Model: 309585, 309657, or specified equivalent). A Becton Dickenson Precision Glide 22 gauge 1.5 inch injection needle (BD Model: 305156 or specified equivalent) is attached to the syringe. The syringe and needle are inserted into the sterile cartridge body. Upon insertion of the syringe and needle, tabs located on the cartridge body retain it in place to prevent accidental exposure to the needle and facilitate safe disposal.

To accommodate differences in skin thickness, a slidable depth control gauge allows adjustment of injection depth to one of three settings: 12, 17, or 22 millimeters from the skin surface. Prior to device activation, the electrodes and injection needle remain recessed within the sterile cartridge body. A plastic safety cap located on the tip of the cartridge protects the operator from accidental stick injury and ensures that the electrodes remain sterile prior to administration.

8.1.5.2 Integrated Applicator

The Integrated Applicator is a reusable, hand-held electromechanical device that contains mechanisms to deploy the electrodes and injection needle into the target muscle tissue and administer the agent of interest. The device is configured so that activation of the device allows the entire procedure to be applied in an automated fashion. As a result, the administration parameters (including the site of agent injection relative to EP application, rate of agent injection, and time interval between agent injection and EP application) will be implemented in a uniform fashion for each subject.

8.1.5.3 Pulse Stimulator

The Integrated Applicator is connected to the Pulse Stimulator through an incorporated cable. The Pulse Stimulator controls the administration sequence, generates the electrical signals necessary to enhance the intracellular delivery of the agent, and monitors the administration sequence for safety hazards. It is an electronic device that is compatible with 120 V/60Hz or 240 V/50Hz supply. The EP conditions used in this study will be applied at an amplitude of 200 V and a total duration of 40 mS, with a 10% duty cycle (e.g., 40 mS of active voltage application within a 400 mS period). The Pulse Stimulator performs a comprehensive self diagnostic upon start-up to ensure that the device and all internal safety systems are functioning properly before an administration procedure can be initiated. In the event of a problem, the user will be notified by the display of an error code on the digital display located on the front of the unit.

8.2 Shipment and Storage

Authorization to ship the Investigational Products to the CRC will be provided in writing by the Sponsor, upon confirmation that all required critical documents for shipment authorization are completed. The Investigational Products will be shipped maintaining the required storage conditions and stored in a secure location in the clinical site’s pharmacy.

HIV-MAG will be stored at 2-8°C
GENEVAX® IL-12 will be stored 2-8°C
Ad35-GRIN/ENV will be stored at -70°C or below
Sodium Chloride Injection, USP 0.9% (placebo) will be stored at 20-25°C
TDS-IM device components will be stored at room temperature

Each of the investigational products will have compliant primary labels affixed onto the vials that will contain unique lot numbers, storage temperature and a US cautionary statement.

8.3 Preparation of Investigational Product (IP)

Detailed instruction will be provided to the pharmacist for preparing each of the investigational products. The pharmacist will not be blinded, but the study physician administering the vaccine will be blinded. Injections should be given within 4 hours of preparation. Final volume for administration is in Table 8.1.4.-1. Instructions for storing used vials until the end of the study and subsequent disposal will be provided. Syringes or other components in direct contact with investigational products will be disposed of in a biohazard container and incinerated or autoclaved.

For details of preparation of vaccines, please see Pharmacy Instructions in the SOM.

8.4 Administration of Investigational Product

Investigational Product will be administered according to the Schedule of Procedures (Appendices A-B).

All administrations of HIV-MAG with or without GENEVAX® IL-12 or the respective placebo consist of 2 IM injections, one into each medial deltoid, by *in vivo* electroporation (IM/EP) using the Ichor Medical Systems TriGrid™ Delivery System (TDS-IM). Prior to injection, a skin pinch test will be performed to set the dept adjuster. Details are provided in the SOM.

The preferred site of administration of Ad35-GRIN/ENV is the deltoid muscle of the non-dominant upper arm (for example, injection in the left arm if the volunteer uses mainly the right arm), unless contraindicated for another reason.

Further information on the administration of the Investigational Products is supplied in the SOM.

8.5 Accountability and Disposal of Investigational Product

The unblinded study pharmacist at each site will be responsible for vaccine accountability and preparation. A witness will be required to confirm accuracy of IP preparation. Verification and sign-off sheets will need to be completed and signed-off for each IP preparation by the pharmacist and counter-signed by the witness.

All used vials will be returned to the pharmacy at the end of each vaccination visit. The date, vial allocation number and location of storage of the returned vials will be recorded. The TDS-IM cartridges with the injection needles affixed inside will be placed in a sharps container following delivery.

During the study, the Investigational Product accountability form, the dispensing log and the log of returned vials will be kept and monitored.

At the end of the study, the used and unused vials will be destroyed; destruction will be witnessed, according to IAVI and site-specific SOPs.

9.0 ASSESSMENTS

9.1 Safety Assessments

Data on local and systemic reactogenicity (i.e., solicited AEs) will be collected by structured interview and medical examination. Volunteers will be given a Memory Aid,

which is a tool to assist with collecting reactogenicity data, but will not be saved as a source document. Data on other adverse events will be collected with open-ended questions. All data will be recorded on the appropriate source documents and entered into the study database.

Local and systemic reactogenicity events will be solicited by study staff prior to vaccination and at least 30 minutes post-vaccination, as specified in the Schedule of Procedures (Appendices A-B).

9.1.1 Local reactogenicity

The presence of local reactogenicity will be assessed at the time points specified in the Schedule of Procedures (Appendices A-B).

Pain, tenderness, erythema/skin discoloration, swelling/hardening or thickening will be assessed and graded using Appendix C, Adverse Event Severity Assessment Table, as a guideline.

9.1.2 Systemic reactogenicity

The presence of systemic reactogenicity will be assessed at the time points specified in the Schedule of Procedures (Appendices A-B).

Fever, chills, headache, nausea, vomiting, malaise, arthralgia, myalgia and fatigue will be assessed and graded using Appendix C, Adverse Event Severity Assessment Table, as a guideline.

9.1.3 Vital signs

At the vaccination visits, vital signs (pulse, respiratory rate, blood pressure and temperature) will be measured by study staff prior to vaccination and at least 30 minutes post-vaccination. At the other study visits, vital signs will be assessed at the time points specified in the Schedule of Procedures (Appendices A-B).

9.1.4 Other adverse events

Other adverse events (AEs) will be collected through one month after the last vaccination in all groups. Serious Adverse Events (SAEs) will be collected throughout the entire study period. Open ended questions will be asked at time points according to the Schedule of Procedures (Appendices A-B). All adverse events will be graded using Appendix C, Adverse Event Severity Assessment Table, as a guideline and will be assessed for causality to the IP.

For more information regarding adverse events refer to Section 10.0, Adverse Events.

9.1.5 Concomitant Medications

During the study, information regarding concomitant medications and reasons for their use will be solicited from the study volunteers at each visit and recorded.

Concomitant receipt of Investigational Products, including other HIV vaccines, is prohibited during the study.

If clinically indicated, inactivated/killed/subunit vaccines (non-HIV) and immunoglobulin may be given up to 14 days before study vaccination(s) or 14 days after the most recent study vaccination (following the post-vaccination blood draw). Live-attenuated vaccines may be given 60 days before study vaccination(s) or 60 days after the most recent post-vaccination blood draw. However, the study vaccination(s) should not be given if there are any continuing symptoms from recently administered non-study vaccines. In this situation, the site investigator should consult with the Medical Monitor before administering the next study vaccination.

If the use of a short tapering course (<2 weeks) of oral corticosteroids is required, the study vaccinations may be continued after a 4-week washout period provided that the medical condition requiring this therapy has completely resolved and in the opinion of both, the site investigator and Medical Monitor, the continuation of the study vaccinations will not jeopardize the safety of the volunteer. Volunteers requiring chronic (> 2 weeks) or long term therapy will not receive any further vaccinations but will continue with follow-up visits until the end of the study.

9.1.6 Routine laboratory parameters

Table 9.1.6-1 shows the laboratory parameters that will be measured routinely. These parameters will include haematology, clinical chemistry, immunological assays and urinalysis. The samples for these tests will be collected at the time points indicated in the Schedule of Procedures (Appendices A-B).

Table 9.1.6-1: Laboratory Parameters

Laboratory Parameter	Test
Haematology	Full blood count (haemoglobin, haematocrit, erythrocytes, leucocytes, platelets), differential count
Clinical Chemistry	Liver function tests: aspartate transferase (AST), alanine transferase (ALT);<br>Kidney function: creatinine
Immunology	CD4 and CD8 T cells (absolute count)
Urinalysis	Dipstick test for protein, blood, glucose, ketones, esterase (leukocytes), nitrite.<br>If clinically significant abnormalities (e.g., blood, protein, leucocytes) are found on dipstick test, then further test(s) will be performed (e.g., microscopy, culture)

9.1.7 Specific screening tests:

Volunteers will be screened for Hepatitis B, C and syphilis and will be excluded if:

- positive for Hepatitis B surface antigen (HBsAg)
- positive for antibodies to Hepatitis C virus (HCV)
- confirmed for active syphilis

9.2 Immunogenicity Assessments

9.2.1 Antibody Responses

- Antibodies against HIV proteins will be measured according to time points as indicated on the Schedule of Procedures (Appendices A-B).
  - Binding antibodies to HIV antigens (frequency and magnitude)
  - Neutralizing antibodies to HIV antigens (seropositivity rates and magnitude of antibody titers)
  - Neutralizing antibodies to the Ad35 vector (frequency and magnitude)
- Other functional antibody assays (e.g., ADCC, ADCVI) may be performed at any of the pre-determined time points

9.2.2 Cellular Responses

Immunogenicity assays, including ELISPOT and intracellular cytokine staining (ICS) for monitoring the number of circulating T cells that can be stimulated to produce cytokines and other effector molecules, will be performed at time points indicated in the Schedule of Procedures (Appendices A-B), using peptide pools representing all or a portion of the encoded HIV antigens.

Further exploratory studies will be carried out to look at the breadth of the response via epitope mapping. Vaccine-matched or other HIV peptide sets such as PTE peptides will be used as the first screen. For examination of cross-reactive T cell responses peptides from HIV-subtypes C and D may be used. Vaccine-induced T cell responses may be further characterized for HLA restriction and additional markers on the responding cells, such as markers for memory, activation, function or markers for homing to mucosal tissues. The ability of PBMC to restrict the growth of HIV *in vitro* may be examined using a Virus Inhibition Assay (VIA). The analytical plan for the immunology studies will be developed and an algorithm will be applied to determine which samples and time points are prioritized for exploratory assays.

Additional cellular, humoral and other immunologic assays may be conducted to assess HIV and vector-specific responses.

9.2.3 PBMC, Serum and Plasma Storage

Samples of cryopreserved PBMC, plasma and serum will be stored as indicated in the Schedule of Procedures (Appendices A-B) and may be used for the purposes of standardisation, quality control and for future assays related to HIV vaccine research and development. These samples will be archived and the testing laboratories will be blinded to the volunteer's identity.

9.3 Other Assessments

9.3.1 HLA Typing

Samples for HLA typing will be collected as specified in the Schedule of Procedures (Appendices A-B) and may be analyzed as warranted.

9.3.2 Serum Antibodies against Human IL-12

Samples for anti-IL12 antibodies will be collected as specified in the Schedule of Procedures (Appendices A-B) and may be analyzed as warranted.

9.3.3 HIV test

Samples will be tested at the time points indicated in the Schedule of Procedures (Appendices A-B). Further information is specified in Section 11.1 HIV Testing.

9.3.4 Pregnancy Test

A urine pregnancy test for all female volunteers will be performed by measurement of Human Chorionic Gonadotrophin (βHCG) at time points indicated in the Schedule of Procedures (Appendices A-B). The results of the pregnancy test must be available and negative prior to each vaccination.

9.3.5 Screening Assessment

A Screening Assessment will be administered at the Screening Visit.

9.3.6 HIV Risk Assessment

Study staff will assess volunteers for their past and current risk of acquiring HIV at time points indicated in Schedule of Procedures (Appendices A and B)

9.3.7 Electroporation Tolerability Assessment

An Electroporation Tolerability Assessment Tool will be provided to the volunteers at each EP administration time point to complete immediately after the procedure, as indicated in Schedule of Procedures (Appendices A and B)

9.3.8 Social Impact Assessment

Each volunteer will have a social impact assessment administered at the time points specified in the Schedule of Procedures (Appendices A and B). This assessement is intended to assess the impact of trial participation on the volunteer’s daily life, if any.

10.0 ADVERSE EVENTS

10.1 Definition

An adverse event (AE) is any untoward medical occurrence in a volunteer administered Investigational Product and which does not necessarily have a causal relationship with the Investigational Product. An AE can therefore be any unfavourable or unintended sign (including an abnormal laboratory finding), symptom, or disease, temporally associated with the use of the Investigational Product whether or not related to the Investigational Product.

10.2 Assessment of Severity of Adverse Events

Assessment of severity of all AEs, including SAEs, is ultimately the responsibility of the Principal Investigator of each site.

The following general criteria should be used in assessing adverse events as mild, moderate, severe or very severe at the time of evaluation:

- Grade 1 (Mild): Symptoms causing no or minimal interference with usual social & functional activities
- Grade 2 (Moderate): Symptoms causing greater than minimal interference with usual social & functional activities
- Grade 3 (Severe): Symptoms causing inability to perform usual social & functional activities
- Grade 4 (Very Severe): Symptoms causing inability to perform basic self-care functions OR Medical or operative intervention indicated to prevent permanent impairment, persistent disability, or death

Guidelines for assessing the severity of specific adverse events and laboratory abnormalities are listed in Appendix C, Adverse Event Severity Assessment Table.

10.3 Relationship to Investigational Product

The relationship of an (S)AE to Investigational Product (IP) is assessed and determined by the Principal Investigator or designee. All medically indicated and available diagnostic methods (e.g., laboratory, blood smear, culture, X-ray, etc…) should be used to assess the nature and cause of the AE/SAE. Best clinical and scientific judgment should be used to assess relationship of AE/SAEs to the IP and/or other cause.

The following should be considered:

- Presence/absence of a clear temporal (time) sequence between administration of the Investigational Product and the onset of AE/SAE

- Presence/absence of another cause that could more likely explain the AE/SAE (concurrent disease, concomitant medication, environmental or toxic factors, etc.)

- Whether or not the AE/SAE follows a known response pattern associated with the Investigational Product

The relationship should be reported as one of the following:

Not Related: clearly explained by another cause (concurrent disease, concomitant medication, environmental or toxic factors, etc.).

Unlikely: more likely explained by another cause (concurrent disease, concomitant medication, environmental or toxic factors, etc.).

Possibly: equally likely explained by another cause but the possibility of the Investigational Product relationship cannot be ruled out (e.g., reasonably well temporally related and/or follows a known Investigational Product response pattern but equally well explained by another cause).

Probably: more likely explained by the Investigational Product (e.g., reasonably well temporally related and/or follows a known Investigational Product response pattern and less likely explained by another cause).

Definitely: clearly related and most likely explained by the Investigational Product.

For the purpose of expedited safety reporting, all possibly, probably or definitely related SAEs are considered Investigational Product-related SAEs.

10.4 Serious Adverse Events

An adverse event is reported as a "Serious Adverse Event" if it meets any the following criteria (as per International Conference on Harmonisation [ICH] Good Clinical Practice [GCP] Guidelines):

- Results in death
- Is life threatening
- Results in persistent or significant disability/incapacity
- Requires in-patient hospitalization or prolongs existing hospitalization
- Is a congenital anomaly/birth defect or spontaneous abortion
- Any other important medical condition that requires medical or surgical intervention to prevent permanent impairment of a body function or structure

Serious Adverse Events (SAEs) should be reported to IAVI within 24 hours of the site becoming aware of the event. All SAEs should be reported using the designated SAE Report Form and sent to the Sponsor as described in the SOM.

To discuss Investigational Product-related SAEs or any urgent medical questions related to the SAE, the site investigator should contact one of the IAVI medical monitors directly (see the Contact List).

The IAVI SAE Report Form should be completed with all the available information at the time of reporting. The minimum data required in reporting an SAE are the volunteer identification number, date of birth, gender, event description (in as much detail as is known at the time), onset date of event (if available), reason event is classified as serious, reporting source (name of Principal Investigator or designee) and relationship to the Investigational Product as assessed by the investigator.

The Principal Investigator or designee is required to write a detailed written report with follow up until resolution or until it is judged by the Principal Investigator or designee to have stabilized. The Principal Investigator or designee must notify the local IRB/IEC of all SAEs as appropriate. In case of Investigational Product-related SAEs, the Sponsor will notify responsible regulatory authorities, Safety Review Board (SRB), and other study sites where the same Investigational Product is being tested.

More details on SAE definitions and reporting requirements are provided in the SOM.

10.5 Clinical Management of Adverse Events

Adverse events (AEs) will be managed by the clinical study team who will assess and treat the volunteer as appropriate, including referral. If any treatment/medical care is required as a result of harm caused by the Investigational Product or study procedures, this will be provided free of charge.

If a volunteer has an AE and/or abnormal laboratory value that is known at the time of study vaccination, the specifications of Section 12.0 will be followed.

Volunteers will be followed until the AE resolves or stabilizes or up to the end of the study, whichever comes last. If at the end of the study, an AE (including clinically significant laboratory abnormality) that is considered possibly, probably or definitely related to the Investigational Product is unresolved, follow-up will continue until resolution if possible and the volunteer will be referred.

10.6 Pregnancy

Although not considered an AE, if a female volunteer becomes pregnant during the study, it is the responsibility of the Principal Investigator or designee to report the pregnancy promptly to IAVI using the designated forms. Vaccinations will be discontinued and the volunteer followed for safety until the end of pregnancy or study completion, whichever occurs last.

Complications of pregnancy that meet criteria for serious specified in Section 10.4 of this Protocol (e.g., eclampsia, spontaneous abortion, etc.) should be reported as SAEs.

If a female volunteer becomes pregnant during the study, then, if possible, approximately 2–4 weeks after delivery, the baby will be examined by a physician to assess its health status and the results will be reported to IAVI.

10.7 Intercurrent HIV Infection

HIV infection cannot be caused by the Investigational Products. If a volunteer acquires HIV through exposure in the community, study vaccinations must be discontinued, and the volunteer should have an Early Termination (ET) visit and offered referral to Protocol H, as described in Section 11.3.2.

Intercurrent HIV infection in study volunteers, although not considered an SAE,must be reported promptly to IAVI using the designated forms. However, medical conditions associated with the HIV infection that meet criteria for serious specified in the Section 10.4 of this Protocol (e.g., sepsis, PCP *pneumonia*, etc.) should be reported as SAEs using the SAE Report Form.

10.8 Serious Event Prior to Investigational Product Administration

If a serious event occurs in the period between the volunteer signing the Informed Consent Form and receiving the first study vaccination, the event will be reported using the SAE form and following the same procedures for SAE reporting, as indicated in Section 10.4. The timing of the event will be indicated by using the relevant checkbox on the SAE form.

11.0 MANAGEMENT OF HIV ISSUES DURING AND FOLLOWING STUDY

11.1 HIV Testing

All Volunteers will be tested for HIV antibodies as indicated in the Schedule of Procedures (Appendices A-B) or as needed, if medical or social circumstances arise. All volunteers will receive HIV risk reduction counselling and pre-HIV-test and post-HIV-test counselling, as specified in Section 11.3.2 Counselling.

A site-specific, predetermined HIV testing algorithm will be followed. In case the routine HIV antibody test is positive, this algorithm will differentiate between an immune response to the vaccine(s) and acquisition of HIV through exposure in the community.

Volunteers who have (a) positive HIV-antibody test(s) as a result of vaccine-induced HIV antibodies will have their test results reported as “Not infected with HIV-1 or HIV-2” (to prevent unblinding of volunteer and staff). A vaccine recipient who still tests HIV positive at the end of the study will be informed of his/her positive test result and offered continuing follow-up until the test becomes negative. If a volunteer is found to be HIV-infected, a newly drawn blood specimen will be collected for confirmation.

Should a volunteer require an HIV test outside the study for personal reasons, it is recommended that the volunteer contact the study staff first. The HIV test can be done at the study site and then processed at an independent laboratory as above. Written evidence of HIV status (HIV-infected or HIV-uninfected) will be provided upon request.

11.2 Social Discrimination as a Result of an Antibody Response to Vaccine

In order to minimize the possibility of social discrimination in volunteers (if any) who develop vaccine-induced HIV antibodies and test positive on a diagnostic HIV antibody test, appropriate diagnostic HIV testing and certification will be provided both during and after the study as needed.

11.3 HIV Infection

Volunteers who are found to be HIV infected at screening (prevalent HIV-infection) and volunteers who acquire HIV infection during the study (intercurrent HIV-infection) will be provided the following:

11.3.1 Counselling

The volunteer will be counselled by the study counsellors. The counselling process will assist the volunteer with the following issues:

- Psychological and social implications of HIV infection
- Whom to inform and what to say
- Implications for sexual partners
- Implications for child-bearing
- Avoidance of transmission to others in future

11.3.2 Referral for Support, Care and Treatment

Volunteers will be referred to a patient support centre or institution of his/her choice for a full discussion of the clinical aspects of HIV infection. Referral will be made to a designated physician or centre for discussion of options of treatment of HIV-infection.

For those individuals who become HIV infected after enrolment in the study (i.e., from first vaccination through final study visit), antiretroviral therapy will be provided when clinically indicated according to accepted treatment guidelines. According to IAVI's Treatment and Care Guidelines, antiretroviral therapy will be provided at no charge for up to 5 years after treatment is initiated, if it is not available through another program.

Volunteers with confirmed HIV infection should have an early termination visit and be asked for their consent to participate in Protocol H. Ideally, the Early Termination (ET) visit and Protocol H study entry visit should occur on the same day.

In the unlikely event that HIV infection is confirmed during a reactogenicity period, contact the medical monitor to determine the course of action on a case by case basis.

HIV-infected pregnant women will be referred for prenatal care and to a program for the Prevention of Mother to Child Transmission (PMTCT) as per site-specific procedures. The pregnant volunteer will be followed according to timeline as specified in Section 10.6.

12. DISCONTINUATION OF VACCINATIONS AND/OR WITHDRAWAL FROM STUDY

12.1 Discontinuation of Vaccinations

Any discontinuation or planned discontinuation from further vaccinations will be discussed with the Sponsor. Volunteers will be discontinued from further vaccination for any of the following reasons:

1. Pregnancy
2. Intercurrent HIV Infection
3. Use of systemic corticosteroids, immunosuppressive, antiviral, anticancer, anti-tuberculosis or other medications considered significant by the investigator. Note: Volunteers requiring chronic corticosteroids (> 2 weeks) or long term therapy will not receive any further vaccinations.
4. A disease or condition or an adverse event that may develop, regardless of relationship to the Investigational Product, if the Principal Investigator or designee is of the opinion that further study vaccinations will jeopardize the safety of the volunteer
5. Anaphylaxis, bronchospasm, laryngeal oedema, convulsions or encephalopathy following study vaccinations
6. Life-threatening adverse event following study vaccination unless not related to the Investigational Product and fully resolved

7. Any immediate hypersensitivity reaction judged to be related to the Investigational Product, if the Principal Investigator or designee is of the opinion that further study vaccinations will jeopardize the safety of the volunteer
8. Volunteer's request to discontinue further vaccination

The following are parameters that require resolution and/or review of clinical history by the Principal Investigator or designee and consultation with the Medical Monitor, prior to continuation of study vaccination(s):

1. Any of the following abnormal laboratory parameters that are known at the time of vaccination:

   Haematology

   - Haemoglobin ≤9.0 g/dL **or** ≤5.55 mmol/L
   - Absolute Neutrophil Count (ANC): $\leq$1000/mm^3 **or** ≤1.0 x 10^9/L
   - Absolute Lymphocyte Count (ALC): ≤500/mm^3 **or** ≤0.5 x 10^9/L
   - Platelets: ≤ 90,000 ≥ 550,000/ mm^3 **or** ≤ 90 x 10^9 ≥ 550 x 10^9/L

   Chemistry

   - Creatinine: >1.4 x ULN
   - AST: ≥2.6 x ULN
   - ALT: ≥2.6 x ULN

2. Receipt of inactivated/killed/subunit vaccines (non-HIV) or immunoglobulin within the previous 14 days or planned receipt within 14 days after vaccination with Investigational Product

3. Receipt of live attenuated vaccines within the previous 60 days or planned receipt within 60 days after vaccination with Investigational Product

4. A Grade 3 or 4 local reactogenicity involving the major part of the injected arm circumference

5. If the use of a short tapering course (<2 weeks) of corticosteroids is required, the study vaccinations may be continued after a 4-week washout period, provided that the medical condition requiring this therapy has completely resolved and in the opinion of both the site investigator and Medical Monitor, the continuation of the study vaccinations will not jeopardize the safety of the volunteer.

12.2. Follow-Up after Discontinuation of Further Vaccinations

Volunteers who have study vaccinations discontinued due to adverse events will be followed until the adverse event resolves or stabilizes or up to the end of the study, whichever comes last. These volunteers will not be replaced.

12.3 Withdrawal from the Study (Early Termination)

Volunteers may be withdrawn from the study permanently for the following reasons:

1. Volunteers may withdraw from the study at any time if they wish, for any reason
2. The Principal Investigator or designee has reason to believe that the volunteer is not complying with the protocol
3. If the Sponsor decides to terminate or suspend the study
4. If a volunteer becomes infected with HIV (through exposure in the community)

If a volunteer withdraws or is withdrawn from the study, all termination visit procedures will be performed according to the Schedule of Procedures (Appendices A-B) where possible. Every effort will be made to determine and document the reason for withdrawal.

13.0 DATA HANDLING

13.1 Data Collection and Record Keeping at the Study Site

Data Collection: All study data will be collected by the clinical study staff using designated source documents and entered onto the appropriate case report forms (CRFs). CRFs will be provided by IAVI and should be handled in accordance with the instructions from IAVI. All study data must be verifiable to the source documentation. A file will be held for each volunteer at the clinic(s) containing all the source documents. Source documentation will be available for review to ensure that the collected data are consistent with the CRFs.

All CRFs and laboratory reports will be reviewed by the clinical team, who will ensure that they are accurate and complete.

Source documents and other supporting documents will be kept in a secure location. Standard GCP practices will be followed to ensure accurate, reliable and consistent data collection.

Source data include but are not limited to:

- Signed Informed Consent Documents
- Dates of visits including dates of vaccinations
- Documentation of any existing conditions or past conditions relevant to eligibility
- Reported laboratory results
- All adverse events
- Concomitant medications
- Local and systemic reactogenicity events

13.2 Data Entry at the Study Site

The data collected at the study site will be recorded on the CRFs by the study staff. All clinical trial information collected will be entered into a database. To provide for real time assessment of safety, data should be entered into the database as soon as reasonably feasible after a visit occurred.

13.3 Data Analysis

The data analysis plan will be developed and agreed upon by the Sponsor, PIs and Collaborating Companies prior to unblinding of the study. The statistician at the Data

Coordinating Centre (DCC), in collaboration with the Principal Investigators, Sponsor, and Collaborating Companies will create tables according to this data analysis plan.

The DCC will conduct the data analysis and will provide interim and final study reports for the Sponsor, Principal Investigator, the SRB and the regulatory authorities, as appropriate.

14.0 STATISTICAL CONSIDERATIONS

14.1 Sample Size

A total of 75 volunteers (60 vaccine/15 placebo) will be enrolled into 5 groups.

14.2 Null Hypothesis

The null hypothesis is based on the primary outcome variables, which are all safety parameters. Let π_0 and π_1 be the proportions of placebo and vaccinated volunteers, respectively, with a primary event. Then the hypothesis is:

$$H_0: \pi_0 = \pi_1 \text{ versus } H_1: \pi_0 < \pi_1$$

i.e., a 1-tailed test of whether the proportion of vaccinated volunteers with an event is greater than the proportion of placebo volunteers with an event.

14.3 Statistical Power and Analysis

Safety and Tolerability:

The rate of local and systemic reactogenicity will be used to assess the differences between vaccine regimens.

The rate of SAEs causally related to the Investigational Product will be used as one measure of the safety of the Investigational Product. AEs that may be temporarily incapacitating (for example, loss or cancellation of work or social activities), which could make an Investigational Product impractical for large scale use if they occur in more than a small proportion of cases, will also be assessed.

All adverse events will be reported, grouped by seriousness, severity and relationship to the Investigational Product (as judged by the investigator).

For any event (e.g., severe AE, SAE related to IP): if none of the volunteers receiving the combination of Investigational Products experiences such an event, then the 2-sided 95% upper confidence limit (CL) for the rate of these events in the population (by the Clopper Pearson method) is 0.06 (n=60) and 0.265 (n=12).

The current sample size will achieve 80% power, at alpha=5%, to detect the following differences in event rates between groups. The calculations are based on Fisher's exact 1-tailed test, using PASS 2008 (www.ncss.com).

Table 14.3-1: Vaccine versus Placebo

# Vols	Rate of events in the placebo group			
**Vaccine/ Placebo**	**1%**	**5%**	**10%**	**20%**
12/3	80%	80%	81%	78%
24/6	53%	57%	59%	59%
60/15	27%	31%	34%	38%

For example, if the true rate of events in placebo recipients (n=15) is 5%, then 60 volunteers receiving vaccine will provide 80% power to detect a statistically significant difference of 31% or more (i.e., a rate of ≥36%).

Table 14.3-2: Comparison of 2 vaccine groups

# Vols	Rate of events in one group			
**per group**	**5%**	**10%**	**20%**	**30%**
12/12	56%	58%	59%	59%
24/12	48%	50%	52%	51%

The current sample size will achieve 80% power, at alpha=5%, to detect the above differences in event rates between individual or combined groups. The calculations are based on Fisher's exact 2-tailed test, using PASS 2008.

One or more descriptive interim analyses of grouped data may be carried out without unblinding the study to investigators or volunteers. At the end of the study, a full analysis will be prepared according to a pre-specified SAP.

Immunogenicity

Cellular immune responses will be analyzed and compared using binomial methods (Fisher's exact test) to examine for the presence or absence of HIV specific T-cell responses quantified by ELISPOT and ICS. Assays will be performed using the IAVI, HIL, and CRC SOPs and standard reagents for all volunteers.

Presence or absence of antibodies to the vaccine-based HIV gene products will be also analyzed. Assays will be performed in a similar fashion in all volunteers.

A descriptive interim analysis may be performed when all volunteers in a given group have reached one month post boost.

15.0 QUALITY CONTROL AND QUALITY ASSURANCE

To ensure the quality and reliability of the data gathered and the ethical conduct of this study, a Study Operations Manual (SOM) will be developed. The SOM includes requirements for reporting and documenting deviations.

Regular monitoring will be performed according to ICH-GCP as indicated in Section 17.3.

An independent audit of the study may be performed by the Sponsor or their designee.

By signing the protocol, the Principal Investigators agree to facilitate study related monitoring, audits, IRB/IEC review and regulatory inspection(s) and direct access to source documents. Such information will be treated as strictly confidential and under no circumstances be made publicly available.

16.0 DATA AND BIOLOGICAL MATERIAL

All data and biological material collected through the study shall be managed in accordance with the Clinical Trial Agreement (CTA). Distribution and use of these data will be conducted by agreement of all parties.

The computerized raw data generated will be held by the DCC on behalf of the Sponsor. The CRC will also hold the final data files and tables generated for the purpose of analysis. The Principal Investigator or designees will have access to the clinical database with appropriate blinding.

17.0 ADMINISTRATIVE STRUCTURE

The Principal Investigator will be responsible for all aspects of the study at the CRC.

17.1 Protocol Safety Review Team

The Protocol Safety Review Team (PSRT) will be formed to monitor the clinical safety data. During the vaccination phase of the trial, the PSRT will review clinical safety data on a weekly basis. An *ad hoc* PSRT review meeting will occur if any of the members of the PSRT requests a special review to discuss a specific safety issue or as specified in the Study Operations Manual.

The PSRT will consist of the IAVI Medical Monitor(s) and PI or designee from each clinical team. An IAVI Medical Monitor will be the PSRT Chair. *Ex officio* members will include the IAVI CMO, IAVI Medical Safety Monitor and representatives from Profectus Biosciences Inc. and Ichor Medical Systems, Inc.

Additional PSRT participants may include the following, as needed:

- Co-investigators and CRC senior clinical research nursing staff
- Laboratory directors
- Data management, study statistician and regulatory staff

The Terms of Reference for PSRT and procedures to be followed are detailed in the SOM.

17.2 Safety Review Board

The Safety Review Board (SRB) will oversee the progress of the study. The SRB will consist of independent clinicians/scientists/statisticians who are not involved in the study. Investigators

responsible for the clinical care of volunteers or representative of the Sponsor may not be a member of the SRB.

However, the SRB may invite the Principal Investigator(s) or designee and a Sponsor representative to an open session of the meeting to provide information on study conduct, present data or to respond to questions.

All safety data will be reviewed by an independent Safety Review Board (SRB) once the first 15 volunteers randomized across all groups have reached the M 2.5 and M 6.5 time points.

17.2.1 Content of Interim Safety Review

The SRB will be asked to review the following data:

- Summary of reactogenicity (i.e., solicited adverse events)
- All clinical adverse events judged by the Principal Investigator or designee to be possibly, probably or definitely related to the Investigational Product
- All laboratory adverse events confirmed on retest and judged by the Principal Investigator or designee to be possibly, probably, or definitely related to Investigational Product and/or clinically significant
- All SAEs

An unblinded presentation of all above-noted events will also be available for the SRB for their review if required by any member of the SRB.

17.3 Criteria for Pausing the Study

Enrolment and vaccinations will be stopped and a safety review conducted by the SRB for any of the following criteria:

- one subject experiences an SAE that is probably or definitely related to the investigational vaccine(s), or two or more subjects experience similar SAEs which are possibly related to the investigational vaccine(s);
- there is a subject death assessed as possibly, probably or definitely related to the investigational vaccine(s)
- one subject experiences injection site ulceration, sterile abscess or necrosis associated with vaccine administration; or
- one subject experiences a severe allergic reaction, such as laryngospasm, bronchospasm or anaphylaxis, associated with vaccine administration.

The Sponsor will request a review by the SRB, or the SRB chair if other SRB members cannot be convened, to be held within 2 business days of the Sponsor learning of the event. The individual volunteer(s)/or study may be unblinded at the discretion of the SRB.

Following this review, the SRB will make a recommendation regarding the continuation or suspension of the vaccinations or the trial and communicate this decision immediately to the Sponsor. The Sponsor then will inform the Principal Investigators without delay.

Additional *ad hoc* review may be specifically requested by the Sponsor, the Principal Investigator(s) or by the SRB.

17.4 Study Supervision

The SRB, the IAVI Chief Medical Officer (CMO) and the IAVI Medical Monitor(s) have access to progress report(s) of this study. Close cooperation will be necessary to track study progress, respond to queries about proper study implementation and management, address issues in a timely manner, and assure consistent documentation, and share information effectively. Rates of accrual, retention, and other parameters relevant to the site's performance will be regularly and closely monitored by the study team, as well as the SRB.

17.5 Study Monitoring

On-site monitoring will be conducted to ensure that the study is conducted in compliance with human subjects' protection and other research regulations and guidelines, recorded and reported in accordance with the protocol, is consistent with SOPs, GCP, applicable regulatory requirements and locally accepted practices. The monitor will confirm the quality and accuracy of data at the site by validation of CRFs against the source documents, such as clinical records. The investigators, as well as volunteers through consenting to the study, agree that the monitor may inspect study facilities and source records (e.g., informed consent forms, clinic and laboratory records, other source documents), as well as observe the performance of study procedures. Such information will be treated as strictly confidential and will under no circumstances be made publicly available.

The monitoring will adhere to GCP guidelines. The Principal Investigator will permit inspection of the facilities and all study-related documentation by authorized representatives of IAVI, and Government and Regulatory Authorities responsible for this study.

17.6 Investigator's Records

Study records include administrative documentation—including reports and correspondence relating to the study—as well as documentation related to each volunteer screened and/or enrolled in the study—including informed consent forms, case report forms, and all other source documents. The investigator will maintain and store, in a secure manner, complete, accurate, and current study records for a minimum of 2 years after marketing application approval or the study is discontinued and applicable national and local health authorities are notified. IAVI will notify the Principal Investigator of these events.

18.0 INDEMNITY

The Sponsor and Institution are responsible to have appropriate liability insurance. For research-related injuries and/or medical problems determined to result from receiving the Investigational Product, treatment including necessary emergency treatment and proper follow-up care will be made available to the volunteer free of charge at the expense of the Sponsor.

19.0 PUBLICATION

A primary manuscript describing safety and immune responses in this trial will be prepared promptly after the data analysis is available. Authors will be representatives of each CRC, the

statistical centre, the laboratories and IAVI, subject to the generally accepted criteria of contributions to the design, work, analysis and written report of the study. Manuscripts will be reviewed by representatives of each participating group, as specified in the CTA.

20.0 ETHICAL CONSIDERATIONS

The Principal Investigator will ensure that the study is conducted in compliance with the protocol, SOPs in accordance with guidelines laid down by the ICH for GCP in clinical studies, the ethical principles that have their origins in the Declaration of Helsinki and applicable local standards and regulatory requirements. In addition to IEC/IRB and regulatory approvals, all other required approvals will be obtained before recruitment of volunteers, as applicable for individual sites.

APPENDIX A: SCHEDULE OF PROCEDURES GROUPS 1-3

Study Month		M0				M1				M2				M3	M5	M6				M7	M9	M12
**Week**		W0		W1	W2	W4				W8		W9	W10	W12	W20	W24		W25	W26	W28	W36	W48 or ET[1]
**Study Day**	**Screen**	0	D3	D7	D14	D28	D31	D35	D42	D56	D59	D63	D70	D84	D140	D168	D171	D175	D182	D196	D252	D336
**Visit Windows (Days)**		-42	± 1	± 3	± 3	± 3	± 1	± 3	± 3	± 3	± 1	± 3	± 3	± 3	± 3	± 7	± 1	± 3	± 3	± 3	± 7	± 7
**Investigational Product**																						
HIV-MAG + GENEVAX® IL12 Placebo		X				X				X												
Ad35-GRIN/ENV / Placebo																X						
**Consent /Assessments/Counselling**																						
Informed Consent	X																					
Screening Assessment	X																					
HIV Risk Assessment		X																				X
HIV Risk Reduction Counselling	X													X						X		X
Pre-/Post HIV-test Counselling	X													X						X		X
Family Planning Counselling	X	X				X				X						X				X	X	
EP Tolerability Assessment		X				X				X												
Social Impact Assessment																						X
**Clinical Safety Assessments**																						
Comprehensive Medical History	X																					
Interim Medical History		X	X	X	X	X	X	X	X	X	X	X	X	X	X	X	X	X	X	X	X[4]	X[4]
Concomitant Medications	X	X	X	X	X	X	X	X	X	X	X	X	X	X	X	X	X	X	X	X		
General Physical Exam	X																					X
Directed Physical Exam		X	X	X	X	X	X	X	X	X	X	X	X	X	X	X	X	X	X	X	X	
Weight & Height	X																					X
Skin Pinch Test	X																					
Vital Signs	X	X[2]				X[2]				X[2]						X[2]						X
Cervical and Axillary Lymph Nodes	X	X	X	X	X	X	X	X	X	X	X	X	X	X	X	X	X	X	X	X	X	X
Local and Systemic Reactogenicity		X[2]	X	X		X[2]	X	X		X[2]	X	X				X[2]	X	X				

Study Month		M0				M1				M2				M3	M5	M6				M7	M9	M12
**Week**		W0		W1	W2	W4				W8		W9	W10	W12	W20	W24		W25	W26	W28	W36	W48 or ET[1]
**Study Day**	**Screen**	0	D3	D7	D14	D28	D31	D35	D42	D56	D59	D63	D70	D84	D140	D168	D171	D175	D182	D196	D252	D336
**Visit Windows (Days)**		-42	± 1	± 3	± 3	± 3	± 1	± 3	± 3	± 3	± 1	± 3	± 3	± 3	± 3	± 7	± 1	± 3	± 3	± 3	± 7	± 7
Adverse Events		X	X	X	X	X	X	X	X	X	X	X	X	X	X	X	X	X	X	X		
Serious Adverse Events	X	X	X	X	X	X	X	X	X	X	X	X	X	X	X	X	X	X	X	X	X	X
**Laboratory Specimens**																						
HIV test	X													X[3]						X[3]		X[3]
Hep BsAg, anti-HepC, active syphilis	X																					
Haematology (+ plasma storage)	X					X				X				X		X			X	X	X	X
Clinical Chemistry (+ serum storage)	X					X				X				X		X			X	X	X	X
Immunology (CD4, CD8)		X																				X
Urinalysis	X	X				X				X						X						X
Pregnancy Test (all female volunteers)	X	X				X				X						X					X	X
**Immunology Lab**																						
Sample HLA Typing		X																				
PBMCs for Cellular immunogenicity assays (ELISPOT, ICS), B cell response + PBMC storage		X							X	X			X	X		X		X	X	X	X	X
Antibody Immunogenicity responses (HIV, Ad35 binding, neutralizing antibodies, anti-IL-12 Ab)		X							X	X			X	X		X			X	X	X	X
**Other**																						
Refer for IAVI Long Term Follow-Up Protocol																						X
**Total blood volume**	23	89				9			80	98			80	103		98		30	98	103	98	103
**Cumulative blood volume**		112				121			201	299			379	482		580		610	708	811	909	1012

[1] Early Termination
[2] At baseline and at least 30 min post vaccination
[3] If HIV infected, conduct ET visit and request consent for Protocol H. If ET and Protocol H visit occur on same day, do not collect PBMCs for ET visit.
[4]Performed to capture SAEs

APPENDIX B: SCHEDULE OF PROCEDURES GROUPS 4 AND 5

Study Month		M0				M1	M4				M5	M7	M10	M12
**Week**		W0		W1	W2	W4	W16		W17	W18	W20	W28	W40	W48 or ET[1]
**Study Day**	**Screen**	0	D3	D7	D14	D28	D112	D115	D119	D126	D140	D196	D280	D336
**Visit Windows (Days)**		-42	± 1	± 3	± 3	± 3	± 3	± 3	± 3	± 3	± 3	± 7	± 7	± 7
**Investigational Product**														
HIV-MAG + GENEVAX® IL12 / Placebo		X[4]					X[5]							
Ad35-GRIN/ENV / Placebo		X[5]					X[4]							
**Consent /Assessments/Counselling**														
Informed Consent	X													
Screening Assessment	X													
HIV Risk Assessment		X												X
HIV Risk Reduction Counselling	X						X				X			X
Pre-/Post HIV-test Counselling	X						X				X			X
Family Planning Counselling	X	X					X				X	X		
EP Tolerability Assessment		X[4]					X[5]							
Social Impact Assessment														X
**Clinical Safety Assessments**														
Comprehensive Medical History	X													
Interim Medical History		X	X	X	X	X	X	X	X	X	X	X[6]	X[6]	X[6]
Concomitant Medications	X	X	X	X	X	X	X	X	X	X	X			
General Physical Exam	X													X
Directed Physical Exam		X	X	X	X	X	X	X	X	X	X	X	X	
Skin Pinch Test	X													
Weight & Height	X													X
Vital Signs	X	X[2]					X[2]							X
Cervical and Axillary Lymph Nodes	X	X	X	X	X	X	X	X	X	X	X	X	X	X
Local and Systemic Reactogenicity		X[2]	X	X			X[2]	X	X					

**Study Month**		**M0**				**M1**	**M4**				**M5**	**M7**	**M10**	**M12**
**Week**		**W0**		**W1**	**W2**	**W4**	**W16**		**W17**	**W18**	**W20**	**W28**	**W40**	**W48 or ET[1]**
**Study Day**	**Screen**	**0**	**D3**	**D7**	**D14**	**D28**	**D112**	**D115**	**D119**	**D126**	**D140**	**D196**	**D280**	**D336**
**Visit Windows (Days)**		**-42**	**± 1**	**± 3**	**± 3**	**± 3**	**± 3**	**± 3**	**± 3**	**± 3**	**± 3**	**± 7**	**± 7**	**± 7**
Adverse Events		X	X	X	X	X	X	X	X	X	X			
Serious Adverse Events	X	X	X	X	X	X	X	X	X	X	X	X	X	X
**Laboratory Specimens**														
HIV test	X					X[3]					X[3]			X[3]
Hep BsAg, anti-HepC**,** active syphilis	X													
Haematology (+ plasma storage)	X	X			X	X	X				X	X		X
Clinical Chemistry (+ serum storage)	X	X			X	X	X				X	X		X
Immunology (CD4, CD8)		X												X
Urinalysis	X	X					X							X
Pregnancy Test (all female volunteers)	X	X					X				X	X		X
**Immunology Lab**														
Sample HLA Typing		X												
PBMCs for Cellular immunogenicity assays (ELISPOT, ICS), B cell response + PBMC storage		X			X	X	X		X	X	X	X	X	X
Antibody Immunogenicity responses (HIV, Ad35 binding, neutralizing antibodies, anti-IL-12 Ab)		X			X	X	X			X	X	X	X	X
**Other**														
Refer for IAVI Long Term Follow-Up Protocol														X
**Total blood volume**	**23**	**98**			**98**	**103**	**98**		**30**	**80**	**103**	**98**	**80**	**103**
**Cumulative volume**		**121**			**219**	**322**	**420**		**450**	**530**	**633**	**731**	**811**	**914**

[1] = Early Termination
[2] = At baseline and 30min post vaccination
[3] = If HIV infected, conduct ET visit and request consent for Protocol H. If ET and Protocol H visits occur on same day, do not collect PBMCs for ET visit
[4] = Group 4 only
[5] = Group 5 only
[6]= Performed to capture SAEs

APPENDIX C: ADVERSE EVENT SEVERITY ASSESSMENT TABLE

Adapted from: DIVISION OF AIDS TABLE FOR GRADING THE SEVERITY OF ADULT AND PEDIATRIC ADVERSE EVENTS Version 1.0, December, 2004; Clarification AUGUST 2009

The Division of AIDS Table for Grading the Severity of Adult and Pediatric Adverse Events ("DAIDS AE Grading Table") is a descriptive terminology that can be utilized for Adverse Event (AE) reporting. A grading (severity) scale is provided for each AE term.

This clarification of the DAIDS Table for Grading the Severity of Adult and Pediatric AE's provides additional explanation of the DAIDS AE Grading Table and clarifies some of the parameters.

I. Instructions and Clarifications

Grading Adult and Pediatric AEs
The DAIDS AE Grading Table includes parameters for grading both Adult and Pediatric AEs. When a single set of parameters is not appropriate for grading specific types of AEs for both Adult and Pediatric populations, separate sets of parameters for Adult and/or Pediatric populations (with specified respective age ranges) are given in the Table. If there is no distinction in the Table between Adult and Pediatric values for a type of AE, then the single set of parameters listed is to be used for grading the severity of both Adult and Pediatric events of that type.

> Note: In the classification of adverse events, the term "severe" is not the same as "serious." Severity is an indication of the intensity of a specific event (as in mild, moderate, or severe chest pain). The term "serious" relates to a participant/event outcome or action criteria, usually associated with events that pose a threat to a participant's life or functioning.

Estimating Severity Grade for Parameters Not Identified in the Table
In order to grade a clinical AE that is not identified in the DAIDS AE grading table, use the category "Estimating Severity Grade" located on Page 3.

Determining Severity Grade for Parameters "Between Grades"
If the severity of a clinical AE could fall under either one of two grades (e.g., the severity of an AE could be either Grade 2 or Grade 3), select the higher of the two grades for the AE. If a laboratory value that is graded as a multiple of the ULN or LLN falls between two grades, select the higher of the two grades for the AE. For example, Grade 1 is 2.5 x ULN and Grade 2 is 2.6 x ULN for a parameter. If the lab value is 2.53 x ULN (which is between the two grades), the severity of this AE would be Grade 2, the higher of the two grades.

Values Below Grade 1
Any laboratory value that is between either the LLN or ULN and Grade 1 should not be graded.

Determining Severity Grade when Local Laboratory Normal Values Overlap with Grade 1 Ranges
In these situations, the severity grading is based on the ranges in the DAIDS AE Grading Table, even when there is a reference to the local lab LLN.

For example: Phosphate, Serum, Low, Adult and Pediatric > 14 years (Page 70) Grade 1 range is 2.50 mg/dL - < LLN. A particular laboratory's normal range for Phosphate is 2.1 – 3.8 mg/dL. A participant's actual lab value is 2.5. In this case, the value of 2.5 exceeds the LLN for the local lab, but will be graded as Grade 1 per DAIDS AE Grading Table.
.

II. Definitions of terms used in the Table:

Basic Self-care Functions Adult

	Activities such as bathing, dressing, toileting, transfer/movement, continence, and feeding.<br><br>Young Children<br>Activities that are age and culturally appropriate (e.g., feeding self with culturally appropriate eating implement).
LLN	Lower limit of normal
Medical Intervention	Use of pharmacologic or biologic agent(s) for treatment of an AE.
NA	Not Applicable
Operative Intervention	Surgical OR other invasive mechanical procedures.
ULN	Upper limit of normal
Usual Social & Functional Activities	Adult<br>Adaptive tasks and desirable activities, such as going to work, shopping, cooking, use of transportation, pursuing a hobby, etc.<br><br>Young Children<br>Activities that are age and culturally appropriate (e.g., social interactions, play activities, learning tasks, etc.).

PARAMETER	GRADE 1<br>MILD	GRADE 2<br>MODERATE	GRADE 3<br>SEVERE	GRADE 4<br>VERY SEVERE
**ESTIMATING SEVERITY GRADE**				
Clinical adverse event NOT identified elsewhere in this DAIDS AE Grading Table	Symptoms causing no or minimal interference with usual social & functional activities	Symptoms causing greater than minimal interference with usual social & functional activities	Symptoms causing inability to perform usual social & functional activities	Symptoms causing inability to perform basic self-care functions OR Medical or operative intervention indicated to prevent permanent impairment, persistent disability, or death
**SYSTEMIC**				
Acute systemic allergic reaction	Localized urticaria (wheals) with no medical intervention indicated	Localized urticaria with medical intervention indicated OR Mild angioedema with no medical intervention indicated	Generalized urticaria OR Angioedema with medical intervention indicated OR Symptomatic mild bronchospasm	Acute anaphylaxis OR Life-threatening bronchospasm OR laryngeal edema
Chills	Symptoms causing no or minimal interference with usual social & functional activities	Symptoms causing greater than minimal interference with usual social & functional activities	Symptoms causing inability to perform usual social & functional activities	NA
Fatigue<br>Malaise	Symptoms causing no or minimal interference with usual social & functional activities	Symptoms causing greater than minimal interference with usual social & functional activities	Symptoms causing inability to perform usual social & functional activities	Incapacitating fatigue/ malaise symptoms causing inability to perform basic self-care functions
Fever (nonaxillary)	37.7 – 38.6°C	38.7 – 39.3°C	39.4 – 40.5°C	> 40.5°C
Pain (indicate body site)<br>DO NOT use for pain due to injection (See Injection Site Reactions: Injection site pain)<br>See also Headache, Arthralgia, and Myalgia	Pain causing no or minimal interference with usual social & functional activities	Pain causing greater than minimal interference with usual social & functional activities	Pain causing inability to perform usual social & functional activities	Disabling pain causing inability to perform basic self-care functions OR Hospitalization (other than emergency room visit) indicated
Unintentional weight loss	NA	5 – 9% loss in body weight from baseline	10 – 19% loss in body weight from baseline	$\geq$ 20% loss in body weight from baseline OR Aggressive intervention indicated [e.g., tube feeding or total parenteral nutrition (TPN)]

PARAMETER	GRADE 1<br>MILD	GRADE 2<br>MODERATE	GRADE 3<br>SEVERE	GRADE 4<br>VERY SEVERE
**INFECTION**				
Infection (any other than HIV infection)	Localized, no systemic antimicrobial treatment indicated AND Symptoms causing no or minimal interference with usual social & functional activities	Systemic antimicrobial treatment indicated OR Symptoms causing greater than minimal interference with usual social & functional activities	Systemic antimicrobial treatment indicated AND Symptoms causing inability to perform usual social & functional activities OR Operative intervention (other than simple incision and drainage) indicated	Life-threatening consequences (e.g., septic shock)
**INJECTION SITE REACTIONS**				
Injection site pain (pain without touching)<br>Or<br>Tenderness (pain when area is touched)	Pain/tenderness causing no or minimal limitation of use of limb	Pain/tenderness limiting use of limb OR Pain/tenderness causing greater than minimal interference with usual social & functional activities	Pain/tenderness causing inability to perform usual social & functional activities	Pain/tenderness causing inability to perform basic self-care function OR Hospitalization (other than emergency room visit) indicated for management of pain/tenderness
Injection site reaction (localized)				
**Adult > 15 years**	Erythema OR Induration of 5x5 cm – 9x9 cm (or 25 cm^2 – 81cm^2)	Erythema OR Induration OR Edema > 9 cm any diameter (or > 81 cm^2)	Ulceration OR Secondary infection OR Phlebitis OR Sterile abscess OR Drainage	Necrosis (involving dermis and deeper tissue)
**Pediatric ≤ 15 years**	Erythema OR Induration OR Edema present but ≤ 2.5 cm diameter	Erythema OR Induration OR Edema > 2.5 cm diameter but < 50% surface area of the extremity segment (e.g., upper arm/thigh)	Erythema OR Induration OR Edema involving ≥ 50% surface area of the extremity segment (e.g., upper arm/thigh) OR Ulceration OR Secondary infection OR Phlebitis OR Sterile abscess OR Drainage	Necrosis (involving dermis and deeper tissue)
Pruritis associated with injection<br>See also Skin: Pruritis (itching - no skin lesions)	Itching localized to injection site AND Relieved spontaneously or with < 48 hours treatment	Itching beyond the injection site but not generalized OR Itching localized to injection site requiring ≥ 48 hours treatment	Generalized itching causing inability to perform usual social & functional activities	NA
**SKIN – DERMATOLOGICAL**				
Alopecia	Thinning detectable by study participant (or by caregiver for young children and disabled adults)	Thinning or patchy hair loss detectable by health care provider	Complete hair loss	NA

PARAMETER	GRADE 1<br>MILD	GRADE 2<br>MODERATE	GRADE 3<br>SEVERE	GRADE 4<br>VERY SEVERE
Cutaneous reaction – rash	Localized macular rash	Diffuse macular, maculopapular, or morbilliform rash OR Target lesions	Diffuse macular, maculopapular, or morbilliform rash with vesicles or limited number of bullae OR Superficial ulcerations of mucous membrane limited to one site	Extensive or generalized bullous lesions OR Stevens-Johnson syndrome OR Ulceration of mucous membrane involving two or more distinct mucosal sites OR Toxic epidermal necrolysis (TEN)
Hyperpigmentation	Slight or localized	Marked or generalized	NA	NA
Hypopigmentation	Slight or localized	Marked or generalized	NA	NA
Pruritis (itching – no skin lesions)<br>(See also Injection Site Reactions: Pruritis associated with injection)	Itching causing no or minimal interference with usual social & functional activities	Itching causing greater than minimal interference with usual social & functional activities	Itching causing inability to perform usual social & functional activities	NA
**CARDIOVASCULAR**				
Cardiac arrhythmia (general)<br>(By ECG or physical exam)	Asymptomatic AND No intervention indicated	Asymptomatic AND Non-urgent medical intervention indicated	Symptomatic, non-life-threatening AND Non-urgent medical intervention indicated	Life-threatening arrhythmia OR Urgent intervention indicated
Cardiac-ischemia/infarction	NA	NA	Symptomatic ischemia (stable angina) OR Testing consistent with ischemia	Unstable angina OR Acute myocardial infarction
Hemorrhage (significant acute blood loss)	NA	Symptomatic AND No transfusion indicated	Symptomatic AND Transfusion of ≤ 2 units packed RBCs (for children ≤ 10 cc/kg) indicated	Life-threatening hypotension OR Transfusion of > 2 units packed RBCs (for children > 10 cc/kg) indicated
Hypertension				
**Adult > 17 years**<br>(with repeat testing at same visit)	140 – 159 mmHg systolic<br>OR<br>90 – 99 mmHg diastolic	160 – 179 mmHg systolic<br>OR<br>100 – 109 mmHg diastolic	≥ 180 mmHg systolic<br>OR<br>≥ 110 mmHg diastolic	Life-threatening consequences (e.g., malignant hypertension) OR Hospitalization indicated (other than emergency room visit)
**Correction**: in Grade 2 to 160 - 179 from > 160-179 (systolic) and to ≥ 100 -109 from > 100-109 (diastolic) and in Grade 3 to ≥ 180 from > 180 (systolic) and to ≥ 110 from > 110 (diastolic).				
**Pediatric ≤ 17 years**<br>(with repeat testing at same visit)	NA	91st – 94th percentile adjusted for age, height, and gender (systolic and/or diastolic)	≥ 95th percentile adjusted for age, height, and gender (systolic and/or diastolic)	Life-threatening consequences (e.g., malignant hypertension) OR Hospitalization indicated (other than emergency room visit)

PARAMETER		GRADE 1<br>MILD	GRADE 2<br>MODERATE	GRADE 3<br>SEVERE	GRADE 4<br>VERY SEVERE
Hypotension		NA	Symptomatic, corrected with oral fluid replacement	Symptomatic, IV fluids indicated	Shock requiring use of vasopressors or mechanical assistance to maintain blood pressure
Pericardial effusion		Asymptomatic, small effusion requiring no intervention	Asymptomatic, moderate or larger effusion requiring no intervention	Effusion with non-life threatening physiologic consequences OR Effusion with non-urgent intervention indicated	Life-threatening consequences (e.g., tamponade) OR Urgent intervention indicated
Prolonged PR interval					
	**Adult > 16 years**	PR interval<br>0.21 – 0.25 sec	PR interval<br>> 0.25 sec	Type II 2nd degree AV block OR Ventricular pause > 3.0 sec	Complete AV block
	**Pediatric ≤ 16 years**	1st degree AV block (PR > normal for age and rate)	Type I 2nd degree AV block	Type II 2nd degree AV block	Complete AV block
Prolonged QTc					
	**Adult > 16 years**	Asymptomatic, QTc interval 0.45 – 0.47 sec OR Increase interval < 0.03 sec above baseline	Asymptomatic, QTc interval 0.48 – 0.49 sec OR Increase in interval 0.03 – 0.05 sec above baseline	Asymptomatic, QTc interval ≥ 0.50 sec OR Increase in interval ≥ 0.06 sec above baseline	Life-threatening consequences, e.g. Torsade de pointes or other associated serious ventricular dysrhythmia
	**Pediatric ≤ 16 years**	Asymptomatic, QTc interval 0.450 – 0.464 sec	Asymptomatic, QTc interval 0.465 – 0.479 sec	Asymptomatic, QTc interval ≥ 0.480 sec	Life-threatening consequences, e.g. Torsade de pointes or other associated serious ventricular dysrhythmia
Thrombosis/embolism		NA	Deep vein thrombosis AND No intervention indicated (e.g., anticoagulation, lysis filter, invasive procedure)	Deep vein thrombosis AND Intervention indicated (e.g., anticoagulation, lysis filter, invasive procedure)	Embolic event (e.g., pulmonary embolism, life-threatening thrombus)
Vasovagal episode (associated with a procedure of any kind)		Present without loss of consciousness	Present with transient loss of consciousness	NA	NA
Ventricular dysfunction (congestive heart failure)		NA	Asymptomatic diagnostic finding AND intervention indicated	New onset with symptoms OR Worsening symptomatic congestive heart failure	Life-threatening congestive heart failure
**GASTROINTESTINAL**					
Anorexia		Loss of appetite without decreased oral intake	Loss of appetite associated with decreased oral intake without significant weight loss	Loss of appetite associated with significant weight loss	Life-threatening consequences OR Aggressive intervention indicated [e.g., tube feeding or total parenteral nutrition (TPN)]
**Comment:** Please note that, while the grading scale provided for Unintentional Weight Loss may be used as a guideline when grading anorexia, this is not a requirement and should not be used as a substitute for clinical judgment.					

PARAMETER	GRADE 1<br>MILD	GRADE 2<br>MODERATE	GRADE 3<br>SEVERE	GRADE 4<br>VERY SEVERE
Ascites	Asymptomatic	Symptomatic AND Intervention indicated (e.g., diuretics or therapeutic paracentesis)	Symptomatic despite intervention	Life-threatening consequences
Cholecystitis	NA	Symptomatic AND Medical intervention indicated	Radiologic, endoscopic, or operative intervention indicated	Life-threatening consequences (e.g., sepsis or perforation)
Constipation	NA	Persistent constipation requiring regular use of dietary modifications, laxatives, or enemas	Obstipation with manual evacuation indicated	Life-threatening consequences (e.g., obstruction)
Diarrhea				
**Adult and Pediatric ≥ 1 year**	Transient or intermittent episodes of unformed stools OR Increase of ≤ 3 stools over baseline per 24-hour period	Persistent episodes of unformed to watery stools OR Increase of 4 – 6 stools over baseline per 24-hour period	Bloody diarrhea OR Increase of ≥ 7 stools per 24-hour period OR IV fluid replacement indicated	Life-threatening consequences (e.g., hypotensive shock)
**Pediatric < 1 year**	Liquid stools (more unformed than usual) but usual number of stools	Liquid stools with increased number of stools OR Mild dehydration	Liquid stools with moderate dehydration	Liquid stools resulting in severe dehydration with aggressive rehydration indicated OR Hypotensive shock
Dysphagia-Odynophagia	Symptomatic but able to eat usual diet	Symptoms causing altered dietary intake without medical intervention indicated	Symptoms causing severely altered dietary intake with medical intervention indicated	Life-threatening reduction in oral intake
Mucositis/stomatitis (clinical exam)<br>Indicate site (e.g., larynx, oral)<br>See Genitourinary for Vulvovaginitis<br>See also Dysphagia-Odynophagia and Proctitis	Erythema of the mucosa	Patchy pseudomembranes or ulcerations	Confluent pseudomembranes or ulcerations OR Mucosal bleeding with minor trauma	Tissue necrosis OR Diffuse spontaneous mucosal bleeding OR Life-threatening consequences (e.g., aspiration, choking)
Nausea	Transient (< 24 hours) or intermittent nausea with no or minimal interference with oral intake	Persistent nausea resulting in decreased oral intake for 24 – 48 hours	Persistent nausea resulting in minimal oral intake for > 48 hours OR Aggressive rehydration indicated (e.g., IV fluids)	Life-threatening consequences (e.g., hypotensive shock)
Pancreatitis	NA	Symptomatic AND Hospitalization not indicated (other than emergency room visit)	Symptomatic AND Hospitalization indicated (other than emergency room visit)	Life-threatening consequences (e.g., circulatory failure, hemorrhage, sepsis)

PARAMETER	GRADE 1<br>MILD	GRADE 2<br>MODERATE	GRADE 3<br>SEVERE	GRADE 4<br>VERY SEVERE
Proctitis (functional-symptomatic)<br>Also see Mucositis/stomatitis for clinical exam	Rectal discomfort AND No intervention indicated	Symptoms causing greater than minimal interference with usual social & functional activities OR Medical intervention indicated	Symptoms causing inability to perform usual social & functional activities OR Operative intervention indicated	Life-threatening consequences (e.g., perforation)
Vomiting	Transient or intermittent vomiting with no or minimal interference with oral intake	Frequent episodes of vomiting with no or mild dehydration	Persistent vomiting resulting in orthostatic hypotension OR Aggressive rehydration indicated (e.g., IV fluids)	Life-threatening consequences (e.g., hypotensive shock)
**NEUROLOGIC**				
Alteration in personality-behavior or in mood (e.g., agitation, anxiety, depression, mania, psychosis)	Alteration causing no or minimal interference with usual social & functional activities	Alteration causing greater than minimal interference with usual social & functional activities	Alteration causing inability to perform usual social & functional activities	Behavior potentially harmful to self or others (e.g., suicidal and homicidal ideation or attempt, acute psychosis) OR Causing inability to perform basic self-care functions
Altered Mental Status<br>For Dementia, see Cognitive and behavioral/attentional disturbance (including dementia and attention deficit disorder)	Changes causing no or minimal interference with usual social & functional activities	Mild lethargy or somnolence causing greater than minimal interference with usual social & functional activities	Confusion, memory impairment, lethargy, or somnolence causing inability to perform usual social & functional activities	Delirium OR obtundation, OR coma
Ataxia	Asymptomatic ataxia detectable on exam OR Minimal ataxia causing no or minimal interference with usual social & functional activities	Symptomatic ataxia causing greater than minimal interference with usual social & functional activities	Symptomatic ataxia causing inability to perform usual social & functional activities	Disabling ataxia causing inability to perform basic self-care functions
Cognitive and behavioral/attentional disturbance (including dementia and attention deficit disorder)	Disability causing no or minimal interference with usual social & functional activities OR Specialized resources not indicated	Disability causing greater than minimal interference with usual social & functional activities OR Specialized resources on part-time basis indicated	Disability causing inability to perform usual social & functional activities OR Specialized resources on a full-time basis indicated	Disability causing inability to perform basic self-care functions OR Institutionalization indicated
CNS ischemia (acute)	NA	NA	Transient ischemic attack	Cerebral vascular accident (CVA, stroke) with neurological deficit

PARAMETER	GRADE 1<br>MILD	GRADE 2<br>MODERATE	GRADE 3<br>SEVERE	GRADE 4<br>VERY SEVERE
Developmental delay – **Pediatric ≤ 16 years**	Mild developmental delay, either motor or cognitive, as determined by comparison with a developmental screening tool appropriate for the setting	Moderate developmental delay, either motor or cognitive, as determined by comparison with a developmental screening tool appropriate for the setting	Severe developmental delay, either motor or cognitive, as determined by comparison with a developmental screening tool appropriate for the setting	Developmental regression, either motor or cognitive, as determined by comparison with a developmental screening tool appropriate for the setting
Headache	Symptoms causing no or minimal interference with usual social & functional activities	Symptoms causing greater than minimal interference with usual social & functional activities	Symptoms causing inability to perform usual social & functional activities	Symptoms causing inability to perform basic self-care functions OR Hospitalization indicated (other than emergency room visit) OR Headache with significant impairment of alertness or other neurologic function
Insomnia	NA	Difficulty sleeping causing greater than minimal interference with usual social & functional activities	Difficulty sleeping causing inability to perform usual social & functional activities	Disabling insomnia causing inability to perform basic self-care functions
Neuromuscular weakness (including myopathy & neuropathy)	Asymptomatic with decreased strength on exam OR Minimal muscle weakness causing no or minimal interference with usual social & functional activities	Muscle weakness causing greater than minimal interference with usual social & functional activities	Muscle weakness causing inability to perform usual social & functional activities	Disabling muscle weakness causing inability to perform basic self-care functions OR Respiratory muscle weakness impairing ventilation
Neurosensory alteration (including paresthesia and painful neuropathy)	Asymptomatic with sensory alteration on exam or minimal paresthesia causing no or minimal interference with usual social & functional activities	Sensory alteration or paresthesia causing greater than minimal interference with usual social & functional activities	Sensory alteration or paresthesia causing inability to perform usual social & functional activities	Disabling sensory alteration or paresthesia causing inability to perform basic self-care functions
Seizure: (new onset) – **Adult ≥ 18 years**<br>See also Seizure: (known pre-existing seizure disorder)	NA	1 seizure	2 – 4 seizures	Seizures of any kind which are prolonged, repetitive (e.g., status epilepticus), or difficult to control (e.g., refractory epilepsy)

PARAMETER	GRADE 1<br>MILD	GRADE 2<br>MODERATE	GRADE 3<br>SEVERE	GRADE 4<br>VERY SEVERE
Seizure: (known pre-existing seizure disorder)<br>**– Adult ≥ 18 years**<br>For worsening of existing epilepsy the grades should be based on an increase from previous level of control to any of these levels.	NA	Increased frequency of pre-existing seizures (non-repetitive) without change in seizure character OR Infrequent break-through seizures while on stable medication in a previously controlled seizure disorder	Change in seizure character from baseline either in duration or quality (e.g., severity or focality)	Seizures of any kind which are prolonged, repetitive (e.g., status epilepticus), or difficult to control (e.g., refractory epilepsy)
Seizure<br>**– Pediatric < 18 years**	Seizure, generalized onset with or without secondary generalization, lasting < 5 minutes with < 24 hours post ictal state	Seizure, generalized onset with or without secondary generalization, lasting 5 – 20 minutes with < 24 hours post ictal state	Seizure, generalized onset with or without secondary generalization, lasting > 20 minutes	Seizure, generalized onset with or without secondary generalization, requiring intubation and sedation
Syncope (not associated with a procedure)	NA	Present	NA	NA
Vertigo	Vertigo causing no or minimal interference with usual social & functional activities	Vertigo causing greater than minimal interference with usual social & functional activities	Vertigo causing inability to perform usual social & functional activities	Disabling vertigo causing inability to perform basic self-care functions
**RESPIRATORY**				
Bronchospasm (acute)	FEV1 or peak flow reduced to 70 – 80%	FEV1 or peak flow 50 – 69%	FEV1 or peak flow 25 – 49%	Cyanosis OR FEV1 or peak flow < 25% OR Intubation
Dyspnea or respiratory distress				
**Adult ≥ 14 years**	Dyspnea on exertion with no or minimal interference with usual social & functional activities	Dyspnea on exertion causing greater than minimal interference with usual social & functional activities	Dyspnea at rest causing inability to perform usual social & functional activities	Respiratory failure with ventilatory support indicated
**Pediatric < 14 years**	Wheezing OR minimal increase in respiratory rate for age	Nasal flaring OR Intercostal retractions OR Pulse oximetry 90 – 95%	Dyspnea at rest causing inability to perform usual social & functional activities OR Pulse oximetry < 90%	Respiratory failure with ventilatory support indicated
**MUSCULOSKELETAL**				
Arthralgia<br>See also Arthritis	Joint pain causing no or minimal interference with usual social & functional activities	Joint pain causing greater than minimal interference with usual social & functional activities	Joint pain causing inability to perform usual social & functional activities	Disabling joint pain causing inability to perform basic self-care functions

PARAMETER	GRADE 1<br>MILD	GRADE 2<br>MODERATE	GRADE 3<br>SEVERE	GRADE 4<br>VERY SEVERE
Arthritis<br>See also Arthralgia	Stiffness or joint swelling causing no or minimal interference with usual social & functional activities	Stiffness or joint swelling causing greater than minimal interference with usual social & functional activities	Stiffness or joint swelling causing inability to perform usual social & functional activities	Disabling joint stiffness or swelling causing inability to perform basic self-care functions
Bone Mineral Loss				
**Adult ≥ 21 years**	BMD t-score<br>-2.5 to -1.0	BMD t-score < -2.5	Pathological fracture (including loss of vertebral height)	Pathologic fracture causing life-threatening consequences
**Pediatric < 21 years**	BMD z-score<br>-2.5 to -1.0	BMD z-score < -2.5	Pathological fracture (including loss of vertebral height)	Pathologic fracture causing life-threatening consequences
Myalgia<br>(non-injection site)	Muscle pain causing no or minimal interference with usual social & functional activities	Muscle pain causing greater than minimal interference with usual social & functional activities	Muscle pain causing inability to perform usual social & functional activities	Disabling muscle pain causing inability to perform basic self-care functions
Osteonecrosis	NA	Asymptomatic with radiographic findings AND No operative intervention indicated	Symptomatic bone pain with radiographic findings OR Operative intervention indicated	Disabling bone pain with radiographic findings causing inability to perform basic self-care functions
**GENITOURINARY**				
Cervicitis<br>(symptoms)<br>(For use in studies evaluating topical study agents)<br>For other cervicitis see Infection: Infection (any other than HIV infection)	Symptoms causing no or minimal interference with usual social & functional activities	Symptoms causing greater than minimal interference with usual social & functional activities	Symptoms causing inability to perform usual social & functional activities	Symptoms causing inability to perform basic self-care functions
Cervicitis<br>(clinical exam)<br>(For use in studies evaluating topical study agents)<br>For other cervicitis see Infection: Infection (any other than HIV infection)	Minimal cervical abnormalities on examination (erythema, mucopurulent discharge, or friability) OR Epithelial disruption < 25% of total surface	Moderate cervical abnormalities on examination (erythema, mucopurulent discharge, or friability) OR Epithelial disruption of 25 – 49% total surface	Severe cervical abnormalities on examination (erythema, mucopurulent discharge, or friability) OR Epithelial disruption 50 – 75% total surface	Epithelial disruption<br>> 75% total surface
Inter-menstrual bleeding (IMB)	Spotting observed by participant OR Minimal blood observed during clinical or colposcopic examination	Inter-menstrual bleeding not greater in duration or amount than usual menstrual cycle	Inter-menstrual bleeding greater in duration or amount than usual menstrual cycle	Hemorrhage with life-threatening hypotension OR Operative intervention indicated

PARAMETER	GRADE 1<br>MILD	GRADE 2<br>MODERATE	GRADE 3<br>SEVERE	GRADE 4<br>VERY SEVERE
Urinary tract obstruction (e.g., stone)	NA	Signs or symptoms of urinary tract obstruction without hydronephrosis or renal dysfunction	Signs or symptoms of urinary tract obstruction with hydronephrosis or renal dysfunction	Obstruction causing life-threatening consequences
Vulvovaginitis (symptoms)<br>(Use in studies evaluating topical study agents)<br>For other vulvovaginitis see Infection: Infection (any other than HIV infection)	Symptoms causing no or minimal interference with usual social & functional activities	Symptoms causing greater than minimal interference with usual social & functional activities	Symptoms causing inability to perform usual social & functional activities	Symptoms causing inability to perform basic self-care functions
Vulvovaginitis (clinical exam)<br>(Use in studies evaluating topical study agents)<br>For other vulvovaginitis see Infection: Infection (any other than HIV infection)	Minimal vaginal abnormalities on examination OR Epithelial disruption < 25% of total surface	Moderate vaginal abnormalities on examination OR Epithelial disruption of 25 - 49% total surface	Severe vaginal abnormalities on examination OR Epithelial disruption 50 - 75% total surface	Vaginal perforation OR Epithelial disruption > 75% total surface
**OCULAR/VISUAL**				
Uveitis	Asymptomatic but detectable on exam	Symptomatic anterior uveitis OR Medical intervention indicated	Posterior or pan-uveitis OR Operative intervention indicated	Disabling visual loss in affected eye(s)
Visual changes (from baseline)	Visual changes causing no or minimal interference with usual social & functional activities	Visual changes causing greater than minimal interference with usual social & functional activities	Visual changes causing inability to perform usual social & functional activities	Disabling visual loss in affected eye(s)
**ENDOCRINE/METABOLIC**				
Abnormal fat accumulation (e.g., back of neck, breasts, abdomen)	Detectable by study participant (or by caregiver for young children and disabled adults)	Detectable on physical exam by health care provider	Disfiguring OR Obvious changes on casual visual inspection	NA
Diabetes mellitus	NA	New onset without need to initiate medication OR Modification of current medications to regain glucose control	New onset with initiation of medication indicated OR Diabetes uncontrolled despite treatment modification	Life-threatening consequences (e.g., ketoacidosis, hyperosmolar non-ketotic coma)
Gynecomastia	Detectable by study participant or caregiver (for young children and disabled adults)	Detectable on physical exam by health care provider	Disfiguring OR Obvious on casual visual inspection	NA

PARAMETER	GRADE 1<br>MILD	GRADE 2<br>MODERATE	GRADE 3<br>SEVERE	GRADE 4<br>VERY SEVERE
Hyperthyroidism	Asymptomatic	Symptomatic causing greater than minimal interference with usual social & functional activities OR Thyroid suppression therapy indicated	Symptoms causing inability to perform usual social & functional activities OR Uncontrolled despite treatment modification	Life-threatening consequences (e.g., thyroid storm)
Hypothyroidism	Asymptomatic	Symptomatic causing greater than minimal interference with usual social & functional activities OR Thyroid replacement therapy indicated	Symptoms causing inability to perform usual social & functional activities OR Uncontrolled despite treatment modification	Life-threatening consequences (e.g., myxedema coma)
Lipoatrophy<br>(e.g., fat loss from the face, extremities, buttocks)	Detectable by study participant (or by caregiver for young children and disabled adults)	Detectable on physical exam by health care provider	Disfiguring OR Obvious on casual visual inspection	NA

LABORATORY

PARAMETER	GRADE 1<br>MILD	GRADE 2<br>MODERATE	GRADE 3<br>SEVERE	GRADE 4<br>VERY SEVERE
**HEMATOLOGY**	***Standard International Units are listed in italics***			
Absolute CD4+ count<br>**– Adult and Pediatric**<br>**> 13 years**<br>(HIV NEGATIVE ONLY)	300 – 400/mm^3<br>*300 – 400/µL*	200 – 299/mm^3<br>*200 – 299/µL*	100 – 199/mm^3<br>*100 – 199/µL*	< 100/mm^3<br>*< 100/µL*
Absolute lymphocyte count<br>**– Adult and Pediatric**<br>**> 13 years**<br>(HIV NEGATIVE ONLY)	600 – 650/mm^3<br>*0.600 x 10^9 –*<br>*0.650 x 10^9/L*	500 – 599/mm^3<br>*0.500 x 10^9 –*<br>*0.599 x 10^9/L*	350 – 499/mm^3<br>*0.350 x 10^9 –*<br>*0.499 x 10^9/L*	< 350/mm^3<br>*< 0.350 x 10^9/L*
**Comment:** Values in children ≤ 13 years are not given for the two parameters above because the absolute counts are variable.				
Absolute neutrophil count (ANC)				
**Adult and Pediatric,**<br>**> 7 days**	1,000 – 1,300/mm^3<br>*1.000 x 10^9 –*<br>*1.300 x 10^9/L*	750 – 999/mm^3<br>*0.750 x 10^9 –*<br>*0.999 x 10^9/L*	500 – 749/mm^3<br>*0.500 x 10^9 –*<br>*0.749 x 10^9/L*	< 500/mm^3<br>*< 0.500 x 10^9/L*
**Infant**[*†]**, 2 – ≤ 7 days**	1,250 – 1,500/mm^3<br>*1.250 x 10^9 –*<br>*1.500 x 10^9/L*	1,000 – 1,249/mm^3<br>*1.000 x 10^9 –*<br>*1.249 x 10^9/L*	750 – 999/mm^3<br>*0.750 x 10^9 –*<br>*0.999 x 10^9/L*	< 750/mm^3<br>*< 0.750 x 10^9/L*
**Infant**[*†]**, ≤1 day**	4,000 – 5,000/mm^3<br>*4.000 x 10^9 –*<br>*5.000 x 10^9/L*	3,000 – 3,999/mm^3<br>*3.000 x 10^9 –*<br>*3.999 x10^9/L*	1,500 – 2,999/mm^3<br>*1.500 x 10^9 –*<br>*2.999 x 10^9/L*	< 1,500/mm^3<br>*< 1.500 x 10^9/L*
**Comment:** Parameter changed from “Infant, < 1 day” to “Infant, ≤1 day”				
Fibrinogen, decreased	100 – 200 mg/dL<br>*1.00 – 2.00 g/L*<br>OR<br>0.75 – 0.99 x LLN	75 – 99 mg/dL<br>*0.75 – 0.99 g/L*<br>OR<br>0.50 – 0.74 x LLN	50 – 74 mg/dL<br>*0.50 – 0.74 g/L*<br>OR<br>0.25 – 0.49 x LLN	< 50 mg/dL<br>*< 0.50 g/L*<br>OR<br>< 0.25 x LLN<br>OR<br>Associated with gross bleeding

LABORATORY				
**PARAMETER**	**GRADE 1 MILD**	**GRADE 2 MODERATE**	**GRADE 3 SEVERE**	**GRADE 4 VERY SEVERE**
Hemoglobin (Hgb)				
**Comment:** The Hgb values in mmol/L have changed because the conversion factor used to convert g/dL to mmol/L has been changed from 0.155 to 0.6206 (the most commonly used conversion factor). For grading Hgb results obtained by an analytic method with a conversion factor other than 0.6206, the result must be converted to g/dL using the appropriate conversion factor for that lab.				
**Adult and Pediatric ≥ 57 days** (HIV POSITIVE ONLY)	8.5 – 10.0 g/dL<br>*5.24 – 6.23 mmol/L*	7.5 – 8.4 g/dL<br>*4.62–5.23 mmol/L*	6.50 – 7.4 g/dL<br>*4.03–4.61 mmol/L*	< 6.5 g/dL<br>< *4.03 mmol/L*
**Adult and Pediatric ≥ 57 days** (HIV NEGATIVE ONLY)	10.0 – 10.9 g/dL<br>*6.18 – 6.79 mmol/L*<br>OR<br>Any decrease<br>2.5 – 3.4 g/dL<br>*1.58 – 2.13 mmol/L*	9.0 – 9.9 g/dL<br>*5.55 - 6.17 mmol/L*<br>OR<br>Any decrease<br>3.5 – 4.4 g/dL<br>*2.14 – 2.78 mmol/L*	7.0 – 8.9 g/dL<br>*4.34 - 5.54 mmol/L*<br>OR<br>Any decrease<br>≥ 4.5 g/dL<br>> *2.79 mmol/L*	< 7.0 g/dL<br>< *4.34 mmol/L*
**Comment:** The decrease is a decrease from baseline				
**Infant*†, 36 – 56 days** (HIV POSITIVE OR NEGATIVE)	8.5 – 9.4 g/dL<br>*5.24 – 5.86 mmol/L*	7.0 – 8.4 g/dL<br>*4.31 – 5.23 mmol/L*	6.0 – 6.9 g/dL<br>*3.72 – 4.30 mmol/L*	< 6.00 g/dL<br>< *3.72 mmol/L*
**Infant*†, 22 – 35 days** (HIV POSITIVE OR NEGATIVE)	9.5 – 10.5 g/dL<br>*5.87 - 6.54 mmol/L*	8.0 – 9.4 g/dL<br>*4.93 – 5.86 mmol/L*	7.0 – 7.9 g/dL<br>*4.34 – 4.92 mmol/L*	< 7.00 g/dL<br>< *4.34 mmol/L*
**Infant*†, ≤ 21 days** (HIV POSITIVE OR NEGATIVE)	12.0 – 13.0 g/dL<br>*7.42 – 8.09 mmol/L*	10.0 – 11.9 g/dL<br>*6.18 – 7.41 mmol/L*	9.0 – 9.9 g/dL<br>*5.59- 6.17 mmol/L*	< 9.0 g/dL<br>< *5.59 mmol/L*
**Correction**: Parameter changed from “Infant < 21 days” to “Infant ≤ 21 days”				
International Normalized Ratio of prothrombin time (INR)	1.1 – 1.5 x ULN	1.6 – 2.0 x ULN	2.1 – 3.0 x ULN	> 3.0 x ULN
Methemoglobin	5.0 – 10.0%	10.1 – 15.0%	15.1 – 20.0%	> 20.0%
Prothrombin Time (PT)	1.1 – 1.25 x ULN	1.26 – 1.50 x ULN	1.51 – 3.00 x ULN	> 3.00 x ULN
Partial Thromboplastin Time (PTT)	1.1 – 1.66 x ULN	1.67 – 2.33 x ULN	2.34 – 3.00 x ULN	> 3.00 x ULN
Platelets, decreased	100,000 – 124,999/mm^3<br>*100.000 x 10^9 – 124.999 x 10^9/L*	50,000 – 99,999/mm^3<br>*50.000 x 10^9 – 99.999 x 10^9/L*	25,000 – 49,999/mm^3<br>*25.000 x 10^9 – 49.999 x 10^9/L*	< 25,000/mm^3<br>*< 25.000 x 10^9/L*
WBC, decreased	2,000 – 2,500/mm^3<br>*2.000 x 10^9 – 2.500 x 10^9/L*	1,500 – 1,999/mm^3<br>*1.500 x 10^9 – 1.999 x 10^9/L*	1,000 – 1,499/mm^3<br>*1.000 x 10^9 – 1.499 x 10^9/L*	< 1,000/mm^3<br>*< 1.000 x 10^9/L*

LABORATORY				
**PARAMETER**	**GRADE 1 MILD**	**GRADE 2 MODERATE**	**GRADE 3 SEVERE**	**GRADE 4 VERY SEVERE**
**CHEMISTRIES**	***Standard International Units are listed in italics***			
Acidosis	NA	pH < normal, but ≥ 7.3	pH < 7.3 without life-threatening consequences	pH < 7.3 with life-threatening consequences
Albumin, serum, low	3.0 g/dL – < LLN<br>*30 g/L – < LLN*	2.0 – 2.9 g/dL<br>*20 – 29 g/L*	< 2.0 g/dL<br>*< 20 g/L*	NA
Alkaline Phosphatase	1.25 – 2.5 x ULN[†]	2.6 – 5.0 x ULN[†]	5.1 – 10.0 x ULN[†]	> 10.0 x ULN[†]
Alkalosis	NA	pH > normal, but ≤ 7.5	pH > 7.5 without life-threatening consequences	pH > 7.5 with life-threatening consequences
ALT (SGPT)	1.25 – 2.5 x ULN	2.6 – 5.0 x ULN	5.1 – 10.0 x ULN	> 10.0 x ULN
AST (SGOT)	1.25 – 2.5 x ULN	2.6 – 5.0 x ULN	5.1 – 10.0 x ULN	> 10.0 x ULN
Bicarbonate, serum, low	16.0 mEq/L – < LLN<br>*16.0 mmol/L – < LLN*	11.0 – 15.9 mEq/L<br>*11.0 – 15.9 mmol/L*	8.0 – 10.9 mEq/L<br>*8.0 – 10.9 mmol/L*	< 8.0 mEq/L<br>*< 8.0 mmol/L*
**Comment:** Some laboratories will report this value as Bicarbonate (HCO_3) and others as Total Carbon Dioxide (CO_2). These are the same tests; values should be graded according to the ranges for Bicarbonate as listed above.				
Bilirubin (Total)				
**Adult and Pediatric > 14 days**	1.1 – 1.5 x ULN	1.6 – 2.5 x ULN	2.6 – 5.0 x ULN	> 5.0 x ULN
**Infant[*†], ≤ 14 days** (non-hemolytic)	NA	20.0 – 25.0 mg/dL<br>*342 – 428 µmol/L*	25.1 – 30.0 mg/dL<br>*429 – 513 µmol/L*	> 30.0 mg/dL<br>*> 513.0 µmol/L*
**Infant[*†], ≤ 14 days** (hemolytic)	NA	NA	20.0 – 25.0 mg/dL<br>*342 – 428 µmol/L*	> 25.0 mg/dL<br>*> 428 µmol/L*
Calcium, serum, high				
**Adult and Pediatric ≥ 7 days**	10.6 – 11.5 mg/dL<br>*2.65 – 2.88 mmol/L*	11.6 – 12.5 mg/dL<br>*2.89 – 3.13 mmol/L*	12.6 – 13.5 mg/dL<br>*3.14 – 3.38 mmol/L*	> 13.5 mg/dL<br>*> 3.38 mmol/L*
**Infant[*†], < 7 days**	11.5 – 12.4 mg/dL<br>*2.88 – 3.10 mmol/L*	12.5 – 12.9 mg/dL<br>*3.11 – 3.23 mmol/L*	13.0 – 13.5 mg/dL<br>*3.245 – 3.38 mmol/L*	> 13.5 mg/dL<br>*> 3.38 mmol/L*
Calcium, serum, low				
**Adult and Pediatric ≥ 7 days**	7.8 – 8.4 mg/dL<br>*1.95 – 2.10 mmol/L*	7.0 – 7.7 mg/dL<br>*1.75 – 1.94 mmol/L*	6.1 – 6.9 mg/dL<br>*1.53 – 1.74 mmol/L*	< 6.1 mg/dL<br>*< 1.53 mmol/L*
**Infant[*†], < 7 days**	6.5 – 7.5 mg/dL<br>*1.63 – 1.88 mmol/L*	6.0 – 6.4 mg/dL<br>*1.50 – 1.62 mmol/L*	5.50 – 5.90 mg/dL<br>*1.38 – 1.51 mmol/L*	< 5.50 mg/dL<br>*< 1.38 mmol/L*
**Comment:** Do not adjust Calcium, serum, low or Calcium, serum, high for albumin				

LABORATORY				
**PARAMETER**	**GRADE 1 MILD**	**GRADE 2 MODERATE**	**GRADE 3 SEVERE**	**GRADE 4 VERY SEVERE**
Cardiac troponin I (cTnI)	NA	NA	NA	Levels consistent with myocardial infarction or unstable angina as defined by the manufacturer
Cardiac troponin T (cTnT)	NA	NA	NA	≥ 0.20 ng/mL<br>OR<br>Levels consistent with myocardial infarction or unstable angina as defined by the manufacturer
Cholesterol (fasting)				
**Adult ≥ 18 years**	200 – 239 mg/dL<br>*5.18 – 6.19 mmol/L*	240 – 300 mg/dL<br>*6.20 – 7.77 mmol/L*	> 300 mg/dL<br>*> 7.77 mmol/L*	NA
**Pediatric < 18 years**	170 – 199 mg/dL<br>*4.40 – 5.15 mmol/L*	200 – 300 mg/dL<br>*5.16 – 7.77 mmol/L*	> 300 mg/dL<br>*> 7.77 mmol/L*	NA
Creatine Kinase	3.0 – 5.9 x ULN†	6.0 – 9.9 x ULN†	10.0 – 19.9 x ULN†	≥ 20.0 x ULN†
Creatinine	1.1 – 1.3 x ULN†	1.4 – 1.8 x ULN†	1.9 – 3.4 x ULN†	≥ 3.5 x ULN†

LABORATORY				
**PARAMETER**	**GRADE 1<br>MILD**	**GRADE 2<br>MODERATE**	**GRADE 3<br>SEVERE**	**GRADE 4<br>VERY SEVERE**
Glucose, serum, high				
Nonfasting	116 – 160 mg/dL<br>*6.44 – 8.88 mmol/L*	161 – 250 mg/dL<br>*8.89 – 13.88 mmol/L*	251 – 500 mg/dL<br>*13.89 – 27.75 mmol/L*	> 500 mg/dL<br>*> 27.75 mmol/L*
Fasting	110 – 125 mg/dL<br>*6.11 – 6.94 mmol/L*	126 – 250 mg/dL<br>*6.95 – 13.88 mmol/L*	251 – 500 mg/dL<br>*13.89 – 27.75 mmol/L*	> 500 mg/dL<br>*> 27.75 mmol/L*
Glucose, serum, low				
**Adult and Pediatric ≥ 1 month**	55 – 64 mg/dL<br>*3.05 – 3.55 mmol/L*	40 – 54 mg/dL<br>*2.22 – 3.06 mmol/L*	30 – 39 mg/dL<br>*1.67 – 2.23 mmol/L*	< 30 mg/dL<br>*< 1.67 mmol/L*
**Infant*†, < 1 month**	50 – 54 mg/dL<br>*2.78 – 3.00 mmol/L*	40 – 49 mg/dL<br>*2.22 – 2.77 mmol/L*	30 – 39 mg/dL<br>*1.67 – 2.21 mmol/L*	< 30 mg/dL<br>*< 1.67 mmol/L*
Lactate	ULN - < 2.0 x ULN without acidosis	≥ 2.0 x ULN without acidosis	Increased lactate with pH < 7.3 without life-threatening consequences	Increased lactate with pH < 7.3 with life-threatening consequences
**Comment:** Added ULN to Grade 1 parameter				
LDL cholesterol (fasting)				
**Adult ≥ 18 years**	130 – 159 mg/dL<br>*3.37 – 4.12 mmol/L*	160 – 190 mg/dL<br>*4.13 – 4.90 mmol/L*	≥ 190 mg/dL<br>*≥ 4.91 mmol/L*	NA
**Pediatric > 2 - < 18 years**	110 – 129 mg/dL<br>*2.85 – 3.34 mmol/L*	130 – 189 mg/dL<br>*3.35 – 4.90 mmol/L*	≥ 190 mg/dL<br>*≥ 4.91 mmol/L*	NA
Lipase	1.1 – 1.5 x ULN	1.6 – 3.0 x ULN	3.1 – 5.0 x ULN	> 5.0 x ULN
Magnesium, serum, low	1.2 – 1.4 mEq/L<br>*0.60 – 0.70 mmol/L*	0.9 – 1.1 mEq/L<br>*0.45 – 0.59 mmol/L*	0.6 – 0.8 mEq/L<br>*0.30 – 0.44 mmol/L*	< 0.60 mEq/L<br>*< 0.30 mmol/L*
Pancreatic amylase	1.1 – 1.5 x ULN	1.6 – 2.0 x ULN	2.1 – 5.0 x ULN	> 5.0 x ULN
Phosphate, serum, low				
**Adult and Pediatric > 14 years**	2.5 mg/dL – < LLN<br>*0.81 mmol/L – < LLN*	2.0 – 2.4 mg/dL<br>*0.65 – 0.80 mmol/L*	1.0 – 1.9 mg/dL<br>*0.32 – 0.64 mmol/L*	< 1.00 mg/dL<br>*< 0.32 mmol/L*
**Pediatric 1 year – 14 years**	3.0 – 3.5 mg/dL<br>*0.97 – 1.13 mmol/L*	2.5 – 2.9 mg/dL<br>*0.81 – 0.96 mmol/L*	1.5 – 2.4 mg/dL<br>*0.48 – 0.80 mmol/L*	< 1.50 mg/dL<br>*< 0.48 mmol/L*
**Pediatric < 1 year**	3.5 – 4.5 mg/dL<br>*1.13 – 1.45 mmol/L*	2.5 – 3.4 mg/dL<br>*0.81 – 1.12 mmol/L*	1.5 – 2.4 mg/dL<br>*0.48 – 0.80 mmol/L*	< 1.50 mg/dL<br>*< 0.48 mmol/L*
Potassium, serum, high	5.6 – 6.0 mEq/L<br>*5.6 – 6.0 mmol/L*	6.1 – 6.5 mEq/L<br>*6.1 – 6.5 mmol/L*	6.6 – 7.0 mEq/L<br>*6.6 – 7.0 mmol/L*	> 7.0 mEq/L<br>*> 7.0 mmol/L*
Potassium, serum, low	3.0 – 3.4 mEq/L<br>*3.0 – 3.4 mmol/L*	2.5 – 2.9 mEq/L<br>*2.5 – 2.9 mmol/L*	2.0 – 2.4 mEq/L<br>*2.0 – 2.4 mmol/L*	< 2.0 mEq/L<br>*< 2.0 mmol/L*
Sodium, serum, high	146 – 150 mEq/L<br>*146 – 150 mmol/L*	151 – 154 mEq/L<br>*151 – 154 mmol/L*	155 – 159 mEq/L<br>*155 – 159 mmol/L*	≥ 160 mEq/L<br>*≥ 160 mmol/L*
Sodium, serum, low	130 – 135 mEq/L<br>*130 – 135 mmol/L*	125 – 129 mEq/L<br>*125 – 129 mmol/L*	121 – 124 mEq/L<br>*121 – 124 mmol/L*	≤ 120 mEq/L<br>*≤ 120 mmol/L*

Triglycerides (fasting)	NA	500 – 750 mg/dL<br>*5.65 – 8.48 mmol/L*	751 – 1,200 mg/dL<br>*8.49 – 13.56 mmol/L*	> 1,200 mg/dL<br>*> 13.56 mmol/L*

LABORATORY				
**PARAMETER**	**GRADE 1<br>MILD**	**GRADE 2<br>MODERATE**	**GRADE 3<br>SEVERE**	**GRADE 4<br>VERY SEVERE**
Uric acid	7.5 – 10.0 mg/dL<br>*0.45 – 0.59 mmol/L*	10.1 – 12.0 mg/dL<br>*0.60 – 0.71 mmol/L*	12.1 – 15.0 mg/dL<br>*0.72 – 0.89 mmol/L*	> 15.0 mg/dL<br>*> 0.89 mmol/L*
**URINALYSIS**	***Standard International Units are listed in italics***			
Hematuria (microscopic)	6 – 10 RBC/HPF	> 10 RBC/HPF	Gross, with or without clots OR with RBC casts	Transfusion indicated
Proteinuria, random collection	1 +	2 – 3 +	4 +	NA
Proteinuria, 24 hour collection				
**Adult and Pediatric<br>≥ 10 years**	200 – 999 mg/24 h<br>*0.200 – 0.999 g/d*	1,000 – 1,999 mg/24 h<br>*1.000 – 1.999 g/d*	2,000 – 3,500 mg/24 h<br>*2.000 – 3.500 g/d*	> 3,500 mg/24 h<br>*> 3.500 g/d*
**Pediatric > 3 mo -<br>< 10 years**	201 – 499 mg/m^2/24 h<br>*0.201 – 0.499 g/d*	500 – 799 mg/m^2/24 h<br>*0.500 – 0.799 g/d*	800 – 1,000 mg/m^2/24 h<br>*0.800 – 1.000 g/d*	> 1,000 mg/ m^2/24 h<br>*> 1.000 g/d*

References

[1] UNAIDS. Report on the global HIV/AIDS epidemic year 2010. www.unaids.org.

[2] Kim JH, Rerks-Ngarm S, Excler JL, Michael NL. HIV vaccines: lessons learned and the way forward. Curr Opin HIV AIDS 2010;5:428-34.

[3] Koff WC. Accelerating HIV vaccine development. Nature 2010; 464:161-2.

[4] McElrath, M. J. and B. F. Haynes (2010). "Induction of Immunity to Human Immunodeficiency Virus Type-1 by Vaccination." Immunity 33(4): 542-554.

[5] Rerks-Ngarm S., P. Pitisuttithum, S. Nitayaphan, J. Kaewkungwal, J. Chiu, R. Paris, et al (2009). Vaccination with ALVAC and AIDSVAX to prevent HIV -1 infection in Thailand. *N Engl J Med*, 361 (23), 2209-20

[6] Walker BD, Burton DR. Towards an AIDS vaccine. Science 2008;320:760-4.

[7] Barouch DH. Challenges in the development of an HIV-1 vaccine. Nature 2008;455:613-9.

[8] Sodora DL, Allan JS, Apetrei C, Brenchley JM, Douek DC, Else JG, *et al.* Toward an AIDS vaccine: lessons from natural simian immunodeficiency virus infections of African nonhuman primate hosts. *Nat Med* 2009;15:861-5.

[9] Johnston MI, Fauci AS. An HIV vaccine – Challenges and Prospects. *N Engl J Med* 2008;359:888-90.

[10] Kaufman DR, Barouch DH. Translational minireview series on vaccines for HIV: T lymphocyte trafficking and vaccine-elicited mucosal immunity. *Clinical and Experimental Immunology 2009;* 157: 165–173.

[11] Franchini G. Choosing the right memory T cell for HIV. *Nature Med* 2009;15:244-6.

[12] Masopust D. Developing an HIV cytotoxic T-lymphocyte vaccine: issues of CD8 T-cell quantity, quality and location. *J Intern Med* 2008;265:125–137.

[13] Coutsinos Z, Absi Z, Henin Y, Guillet JG, Launay O. Designing an effective AIDS vaccine: strategies and current status. *Rev Med Interne* 2008;29:632-41.

[14] Mascola, J. R., G. Stiegler, T. C. VanCott, H. Katinger, C. B. Carpenter, C. E. Hanson, H. Beary, D. Hayes, S. S. Frankel, D. L. Birx, and M. G. Lewis. Protection of macaques against vaginal transmission of a pathogenic HIV-1/SIV chimeric virus by passive infusion of neutralizing antibodies. *Nat Med* 2000;6:207-10.

[15] Pantophlet R, Burton DR. GP120: target for neutralizing HIV-1 antibodies. *Annu Rev Immunol* 2006;24:739-69.

[16] Hessell AJ, Poignard P, Hunter M, Hangartner L, Tehrani DM, Bleeker WK, *et al.* Effective, low-titer antibody protection against low-dose repeated mucosal SHIV challenge in macaques. *Nat Med* 2009;15:951-4.

[17] Zhang MY, Dimitrov DS. Novel approaches for identification of broadly cross-reactive HIV-1 neutralizing human monoclonal antibodies and improvement of their potency. *Curr Pharm Des* 2007;13:203-12.

[18] Montero M, van Houten NE, Wang X, Scott JK. The membrane-proximal external region of the human immunodeficiency virus type 1 envelope: dominant site of antibody neutralization and target for vaccine design. *Microbiol Mol Biol Rev* 2008;72:54-84.

[19] Burton DR, Desrosiers RC, Johnson PR, Koff WC. An AIDS vaccine: no time to give up. *Lancet* 2004;364:1938.

[20] Montefiori D, Sattentau Q, Flores J, Esparza J, Mascola J; Working Group convened by the Global HIV Vaccine Enterprise. Antibody-based HIV-1 vaccines: recent developments and future directions. *PLoS Med* 2007;4:e348.

[21] Borrow P, Lewicki H, Hahn BH, Shaw GM, Oldstone MB. Virus-specific CD8+ cytotoxic T lymphocyte activity associated with control of viremia in primary human immunodeficiency virus type 1 infection. *J Virol 1994;* 68: 6103–10.

[22] Koup RA, Safrit JT, Cao Y, Andrews CA, McLeod G, *et al.* Temporal association of cellular immune responses with the initial control of viremia in primary human immunodeficiency virus type 1 syndrome. *J Virol* 1994; 68: 4650–5.

[23] Burgers WA, Riou C, Mlotshwa M, Maenetje P, de Assis Rosa D*, et al*. Association of HIV-specific and total CD8+ T memory phenotypes in subtype C HIV-1 infection with viral set point. *J Immunol* 2009; 182:4751-61.

[24] Jin X, Bauer DE, Tuttleton SE, Lewin S, Gettie A, *et al.* Dramatic rise in plasma viremia after CD8+ T cell depletion in simian immunodeficiency virus-infected macaques. *J Exp Med* 1999; 189: 991–8.

[25] Friedrich TC, Valentine LE, Yant LJ, Rakasz EG, Piaskowski SM, *et al*. Subdominant CD8+ T cell responses are involved in durable control of AIDS virus replication. *J Virol* 2007*;* 81: 3465–76.

[26]Watkins DI, Burton DR, Kallas EG, Moore JP, Koff WC. Nonhuman primate models and the failure of the Merck HIV-1 vaccine in humans. *Nat Med* 2008;14:617-21.

[27] Wilson NA, Keele BF, Reed JS, Piaskowski SM, Mac Nair CE, *et al*. Vaccine-induced Cellular Responses Control SIV Replication After Heterologous Challenge*. J Virol* 2009; 83:6508-21.

[28] Scott G. Hansen, Julia C. Ford, Matthew S. Lewis, Abigail B. Ventura, Colette M.Hughes, Lia Coyne-Johnson, Nathan Whizin, Kelli Oswald, Rebecca Shoemaker, Tonya Swanson, Alfred W. Legasse, Maria J. Chiuchiolo, Christopher L. Parks, Michael K. Axthelm, Jay A. Nelson, Michael A. Jarvis, Michael Piatak Jr, Jeffrey D. Lifson & Louis J. Picker. Profound early control of highly pathogenic SIV by an effector memory T-cell vaccine | N AT U R E on line May 2011.

[29] Masopust D. Developing an HIV cytotoxic T-lymphocyte vaccine: issues of CD8 T-cell quantity, quality and location. *J Intern Med* 2008; 265: 125–37.

[30] Sallusto F, Geginat J, Lanzavecchia A. Central memory and effector memory T cell subsets: function, generation, and maintenance. *Annu Rev Immunol* 2004; 22:745-63.

[31] Pahar B, Lackner AA, Piatak M Jr, Lifson JD, Wang X, *et al*. Control of viremia and maintenance of intestinal CD4(+) memory T cells in SHIV(162P3) infected macaques after pathogenic SIV(MAC251) challenge. *Virology* 2009; 387:273-84.

[32] Hess C, Altfeld M, Thomas SY, Addo MM, Rosenberg ES, *et al*. HIV-1 specific CD8+ T cells with an effector phenotype and control of viral replication. *Lancet* 2004; 363:863-6.

[33] Rosenberg ES, Walker BD. HIV type 1-specific helper T cells: a critical host defense. *AIDS Res Hum Retroviruses* 1998; 14(Suppl 2):S143-S147.

[34] Dyer WB, Zaunders JJ, Yuan FF, Wang B, Learmont JC, *et al*. Mechanisms of HIV non-progression; robust and sustained CD4+ T-cell proliferative responses to p24 antigen correlate with control of viremia and lack of disease progression after long-term transfusion-acquired HIV-1 infection. *Retrovirology* 2008; 5:112.

[35] Sacha JB, Giraldo-Vela JP, Buechler MB, Martins MA, Maness NJ, *et al*. Gag- and Nef-specific CD4+ T cells recognize and inhibit SIV replication in infected macrophages early after infection. *Proc Natl Acad Sci USA* 2009; 106:9791-6.

[36] Riddell SR, Greenberg PD. T-Cell therapy of cytomegalovirus and human immunodeficiency virus infection. *Journal of Antimicrobial Chemotherapy*; 2000; 45: 35-45

[37] Lichterfeld M, Kaufmann DE, Yu XG, Mui SK, Addo MM, *et al*. Loss of HIV-1-specific CD8+ T cell proliferation after acute HIV-1 infection and restoration by vaccine-induced HIV-1-specific CD4+ T cells. *J Exp Med* 2004; 200:701-12.

[38] Woodland DL. Jump-starting the immune system: prime-boosting comes of age. *Trends Immuno* 2004; 25:98-104.

[39] Radosević K, Rodriguez A, Lemckert A, Goudsmit J. Heterologous prime-boost vaccinations for poverty-related diseases: advantages and future prospects. *Expert Rev Vaccines* 2009; 8:577-92.

[40] Paris RM, Kim JH, Robb ML, Michael NL. Prime-boost immunization with poxvirus or adenovirus vectors as a strategy to develop a protective vaccine for HIV-1. Expert Rev Vaccines 2010;9:1055-69.

[41] Xu J, Ren L, Huang X, Qiu C, Liu Y, *et al.* Sequential priming and boosting with heterologous HIV immunogens predominantly stimulated T cell immunity against conserved epitopes. *AIDS* 2006;20:2293-303.

[42] Liu J, O'Brien KL, Lynch DM, Simmons NL, La Porte A, *et al*. Immune control of an SIV challenge by a T-cell-based vaccine in rhesus monkeys. *Nature* 2009;457:87-91.
[43] Keefer M et al. et al. AIDS Vaccine 2010, Atlanta GA, 28 September – 1st October, 2010, AIDS Research and Human Retroviruses. October 2010, 26(10): A-1-A-184.

[44]Aihara H. Miyazaki J. Gene transfer into muscle by electroporation in vivo. Nat Biotechnol. 1998;16(9):867-70.

[45] Gehl J, Mir LM. Determination of optimal parameters for in vivo gene transfer by electroporation, using a rapid in vivo test for cell permeabilization. Biochem Biophys Res Commun. 1999;261(2):377-80.

[46] Vasan S, Hurley A, Schlesinger SJ, Hannaman D, Gardiner DF, et al. 2011 In Vivo Electroporation Enhances the Immunogenicity of an HIV-1 DNA Vaccine Candidate in Healthy Volunteers. PLoS ONE 6(5): e19252. doi:10.1371/journal.pone.0019252

[47] A. Luckay, M. K. Sidhu, R. Kjeken, S. Megati, S.-Yen Chong, V. Roopchand, D. Garcia-Hand, R. Abdullah, R. Braun, D. C. Montefiori, M. Rosati, B. K. Felber, G. N. Pavlakis, I. Mathiesen, Z. R. Israel, J. H. Eldridge and M. A. Egan. Effect of plasmid DNA vaccine design and in vivo electroporation on the resulting vaccine-specific immune responses in rhesus macaques. J. Virol, 81:5257-5269 (2007).

[48] E. B. Schadeck, M. Sidhu, M. A. Egan, S-Y. Chong, P. Piacente, A. Masood, D. Garcia-Hand, S. Cappello, V. Roopchand, S. Megati, J. Quiroz, J. D. Boyer, B. K. Felber, G. N. Pavlakis, D. B. Weiner, J. H. Eldridge and Z. R. Israel. Dose sparing effect by plasmid encoded IL-12 adjuvant on a SIVgag-plasmid DNA vaccine in rhesus macaques. Vaccine, 24:4510-4523 (2006).

[49] R. Xu, S. Megati, V. Roopchand, A. Luckay, A. Masood, D. Garcia-Hand, M. Rosati, D. B. Weiner, B. K. Felber, G. N. Pavlakis, M. K. Sidhu, J. H. Eldridge and M. A. Egan. Comparative ability of various plasmid-based cytokines and chemokines to adjuvant the activity of HIV plasmid DNA vaccines. Vaccine 26:4819-29 (2008).

[50] S. Chong, M. A. Egan, M. Kutzler, S. Megati, A. Masood, D. C. Montefiori, J. Quiroz, M. Rosati, E. B. Schadeck, J. D. Boyer, G. N. Pavlakis, D. B. Weiner, M. Sidhu, J. H. Eldridge and Z. R. Israel. Comparative ability of plasmid IL-12 and IL-15 to enhance cellular and humoral immune responses elicited by a SIVgag plasmid DNA vaccine and alter disease progression following SHIV89.6P challenge in Rhesus macaques. Vaccine, 25:4967-4982 (2007).

[51] M. A. Egan, S. Megati, V. Roopchand, D. Garcia-Hand, A. Luckay, S-Y. Chong, M. Rosati, S. Sackitey, D. B. Weiner, B. K. Felber, G. N. Pavlakis, Z. R. Israel, J. H. Eldridge and M. K. Sidhu. Rational Design of a Plasmid DNA Vaccine Capable of Eliciting Cell-mediated Immune Responses to Multiple HIV Antigens in Mice. Vaccine, 24:4677-4687 (2006).

[52] Vasan S, Hurley A, Schlesinger SJ, Hannaman D, Gardiner DF, et al. 2011 In Vivo Electroporation Enhances the Immunogenicity of an HIV-1 DNA Vaccine Candidate in Healthy Volunteers. PLoS ONE 6(5): e19252. doi:10.1371/journal.pone.0019252

[53] Keefer, M, et al. 2010, September 29. Preliminary Results of Safety and Immunogenicity of Ad35-GRIN/Env HIV Vaccine in HIV-uninfected Subjects (IAVI B001), Oral presentation at AIDS Vaccine 2010, Atlanta, GA.
