# Supplementary material for: Acceptability and tolerability of repeated intramuscular electroporation of Multi-antigenic HIV (HIVMAG) DNA vaccine among healthy African participants in a phase 1 randomized controlled trial
Source: PLoS One. 2020 May 29;15(5):e0233151. doi: 10.1371/journal.pone.0233151 (PMC7259687; doi:10.1371/journal.pone.0233151)
Supplement: Attachment — Submitted filename: Response to Dr. Diemert.doc [file pone.0233151.s005.doc]

Dear Dr. Diemert,

Thank you for being willing to solicit another opinion on our manuscript. We very much appreciate this opportunity, and have responded to the additional reviewer comments below, as you requested. Thank you again for your consideration of our concerns.

**Author issues with prior review:**

As the statistician who designed and conducted this analysis, I stand by our methodological decisions and feel that the reviewer has misunderstood our analysis and our explanations in response to stated concerns about it. Specifically, the reviewer disagrees with our use of GEE techniques to account for the correlations created by inclusion of multiple measurements per person. Although a prior study authored by Dr. Mpendo had looked at subject responses to IM/EP in aggregate, she wished to look at individual responses over time, requiring repeated measures analysis.

We have found the reviewer’s concern that the paper is misleading and potentially harmful to be truly puzzling, and have responded to this concern to the best of our ability.  Although the reviewer has asked us to remove the analysis which is at the heart of this paper, this would repeat analyses already published, and be counter to the purpose of the paper. I do not agree with the Reviewer’s belief that GEE is inappropriate here; GEE merely adjusts the standard errors in a model to include the within person variability that is generated when multiple observations are made on the same person. (And then averages over all observations.)

We thank the editors for allowing further consideration of our manuscript, despite what the prior reviewer feels are fatal flaws. Should you decide not to publish the paper, we will accept your decision and withdraw it.

Respectfully yours,

Drs. Juliet Mpendo, Nancy Hills, Matt Price and Pat Fast

**Second round comments from reviewer and our responses:**

Reviewer #2: Thank you for your thorough and thoughtful consideration of my comments and for clarifying the statistical methods.

That said, I was somewhat perplexed by some of your responses to my comments. My goal as a reviewer, especially a statistical reviewer, is to ensure criteria 3 and 4 of the publication criteria are met. My desire for statistical evidence is based criterion #4, "conclusions are presented in an appropriate fashion and are supported by the data." I am only responding to the statements that you have made in the manuscript. In order to be in accordance with the publication criteria, I am asking for evidence to be presented which supports those claims. My "desire" for empirical evidence is based on the journal's desire for empirical evidence. Of course, I may have misread or misinterpreted certain statements and you should feel free to correct me, but that's where I am coming from.

**Response:** We feel that we have responded to the reviewer’s concerns to the best of our ability.

Second, a technical comment: for GEE models to be robust, there needs to be around 20 groups per condition (<https://doi.org/10.2105/AJPH.94.3.423>). That's because marginal models are trying to make conclusions at the aggregate level and, when there are not many groups (in your case, people), there's been simulation studies that show the results became less robust once the number of groups starts dropping below that level.

**Response:** The subject of the article to which the reviewer refers is actually group-randomized trials; i.e., the group is the unit of analysis. This presents different problems than we address here; our purpose in utilizing GEE is simply to adjust the standard errors in the analysis to account for the added within-person variability created when multiple measurements are made on the same person. While our small sample may not represent the most ideal characteristics for GEE analysis, it certainly does not preclude its use.

In addition, regarding the modeling, you stated in your responses that, "…our primary interest was in determining whether the electroporation process was perceived to be painful enough to contribute to the risk of loss-to-follow-up, or rather was considered acceptable enough to warrant subjects returning twice more after an initial exposure." This sounds to me like pain levels are the explanatory variable and that LTFU/retention is the outcome. If that is the case, then the models presented need to be changed to reflect this.

**Response:** Our primary purpose, as stated, was to examine time as a predictor of reported pain from the electroporation procedure. Since follow-up in our study is perfect, it would not be possible to use retention as an outcome. We have tried to make clear that we believe that the evidence we found supports acceptability of the procedure and retention in the study, and not that it causes it or proves it.

Regarding the Likert-like scale for pain, there is large disagreement on whether such a scale can/should be treated as continuous. I am still not comfortable with this, but there's not much that can be done at this stage other than to say to please try to implement a more nuanced pain scale in future studies.

**Response:** We do not agree with reviewer that a Likert-scale response should be viewed as continuous, for the reasons stated in our prior response.

Though, putting the modeling aside, as I read through your responses and revisions, I still maintain that you are making this manuscript much harder and complex than it needs to be. As I see it, you have two main points: (1) almost all patients rated this procedure as acceptable and (2) all the patients were retained. Those are important points and I agree, even with a small sample size, that these will support your end goal of showing that the acceptability and tolerability of this procedure. But, the modeling convolutes the manuscript and I don't think it needs to be there.

**Response:** Acceptability data *in aggregate* have already been published, as we pointed out. We do not view a model that merely accounts for the correlation between multiple observations on the same person to be complex or convoluted.

Further, again pulling from your comments, I think this is what you really want: "It is rather our intention to summarize the experience of our subjects as they went through three vaccinations."

**Response:** We feel that our methodology has been appropriate to address this issue which necessarily must account for repeated observations per person.
